# More than bad luck: Cancer and aging are linked to replication-driven changes to the epigenome

**DOI:** 10.1126/sciadv.adf4163

**Published:** 2023-07-19

**Authors:** Christopher J. Minteer, Kyra Thrush, John Gonzalez, Peter Niimi, Mariya Rozenblit, Joel Rozowsky, Jason Liu, Mor Frank, Thomas McCabe, Raghav Sehgal, Albert T. Higgins-Chen, Erin Hofstatter, Lajos Pusztai, Kenneth Beckman, Mark Gerstein, Morgan E. Levine

**Affiliations:** ^1^ Department of Pathology, Yale School of Medicine, New Haven, CT, USA.; ^2^ San Diego Institute of Science, Altos Labs, San Diego, CA, USA.; ^3^ Department of Internal Medicine, Section of Medical Oncology, Yale School of Medicine, New Haven, CT, USA.; ^4^ Department of Molecular Biophysics and Biochemistry, Yale University, New Haven, CT, USA.; ^5^ Biomedical Genomics Center, University of Minnesota, Minneapolis, MN, USA.

## Abstract

Aging is a leading risk factor for cancer. While it is proposed that age-related accumulation of somatic mutations drives this relationship, it is likely not the full story. We show that aging and cancer share a common epigenetic replication signature, which we modeled using DNA methylation from extensively passaged immortalized human cells in vitro and tested on clinical tissues. This signature, termed CellDRIFT, increased with age across multiple tissues, distinguished tumor from normal tissue, was escalated in normal breast tissue from cancer patients, and was transiently reset upon reprogramming. In addition, within-person tissue differences were correlated with predicted lifetime tissue-specific stem cell divisions and tissue-specific cancer risk. Our findings suggest that age-related replication may drive epigenetic changes in cells and could push them toward a more tumorigenic state.

## Teaser

Cellular replication leaves an epigenetic fingerprint that may partially underly the age-associated increase in cancer risk.

## INTRODUCTION

Aging is a leading carcinogen, with cancer risk increasing more than 4000% between the ages of 25 and 65 (*1*). This staggering increase is not the entire cancer narrative (*2*), but it is the exigent characteristic of the disease. While some cancers—like those of the bone, brain, or nervous system—are diagnosed at higher frequencies in children and adolescence (*3*), recent studies suggest that only one-third of all cancers are linked to age-independent factors. This begs the question, to what degree is cancer preventable, as opposed to a largely unavoidable outcome of time (*4*, *5*)? This premise, argued by Tomasetti and Vogelstein in 2015, later became known as the bad luck hypothesis (*4*). The controversial theory was based on the idea that the cumulative number of divisions a cell undergoes over time is related to its propensity for tumorigenesis.

Tomasetti and Vogelstein concluded that stochastic mutation accumulation in presumed stem cells explains a far greater number of cancers than do germline predisposition and environmental factors. However, one caveat that should be considered is that their data drew from population-level statistics, rather than the individual-level. Interindividual heterogeneity can be obscured only by measuring trends preserved across the population and tissues. Population-level statistics do measure interindividual differences, but only differences that trend across most of the population. This does not exclude the likely possibility of between-person heterogeneity when considering risk of cancer within a given tissue type. The theory also relies on a presumed tissue-specific stem cell as the originator of cancer, a hypothesis that has been called into question. It is an open question whether variations in rates of biological aging, interpersonal differences in cumulative cell divisions in tissues, and their accompanying molecular changes contribute to differential risk of cancer across individuals.

Mutations are not the only, or perhaps even the most important, molecular events that result from cellular proliferation. We and others have shown that DNA methylation (DNAm) is also substantially altered as a direct function of cell division (*6*–*9*). Further, the epigenome has been shown to undergo dramatic changes with aging and is implicated in establishing, driving, and maintaining many cancers (*10*–*18*). Coincidently, the DNAm changes observed in aging, cancer, and proliferation share some notable patterns. In general, they tend to be characterized by gains in methylation at promoters—especially those marked by polycomb group (PcG) factor targets—and loss of methylation in intergenic regions and repetitive elements (*19*). Thus, one hypothesis is that as cells replicate in aging tissues, they may also take on epigenetic signatures that are more cancer-like, making the leap to oncogenic transformation progressively more likely with time (*20*–*24*). Further, the greater the replication rate in a given tissue, the faster this transformation may occur. Previous work by Klutstein *et al.* (*24*) demonstrated that tissue variation in cancer risk is also correlated with aberrant CpG island methylation, while Duran-Ferrer *et al.* (*23*) used a B cell model to link mitotic-derived methylation variability to B cell tumor outcomes. This evidence and others (*4*, *7*, *8*) point toward a direction of cellular replication–linked methylation playing a key factor in differential cancer risk.

To date, the field has linked (i) age-related epigenetic changes and cancer phenomenon and (ii) replication-related changes to cancer, but little evidence exists linking all three simultaneously. It also remains unclear whether age-related replication-based changes are tumorigenic switches, and can perhaps predate the disease, or whether they are simply outcomes of cancer and uncontrolled proliferation. To test these hypotheses, we quantified a “replication fingerprint” in DNAm data derived from extensively passaged immortalized human cells using a de novo computational training platform, contrary to the common top-down training methods, which are considered a “black box.” We show that this signature accumulates with aging in multiple tissues, is stronger in tumor relative to normal tissues, appears accelerated in the normal tissue of cancer patients, correlates with tissue-specific differences in lifetime cancer risk and total stem cell divisions, and is transiently reset upon reprogramming.

## RESULTS AND DISCUSSION

### Identification and isolation of replication fingerprints

**Fig. 1.**
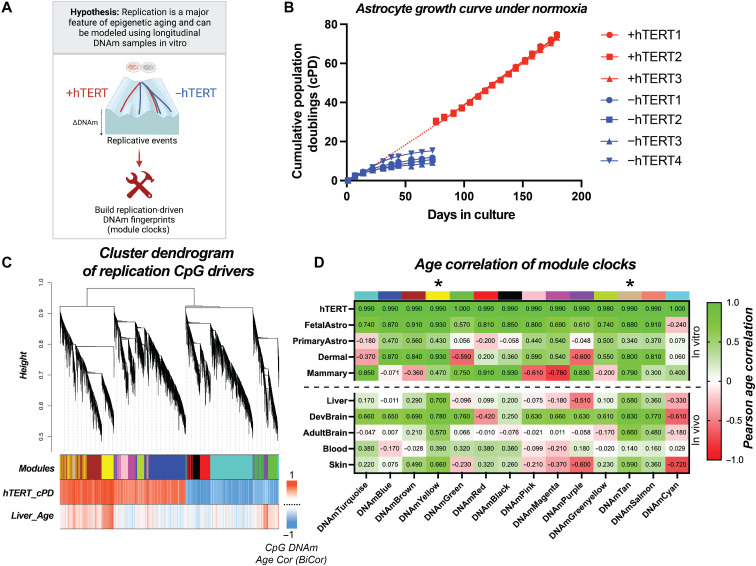
Replication-driven epigenetic fingerprints trained from longitudinal hTERT trajectory in vitro capture multitissue epigenetic aging. (**A**) Schematic displaying goal of cataloging replication aging trajectories using DNAm from a longitudinal hTERT passaging model in vitro. (**B**) Growth curve of human astrocytes under normoxia showing that growth arrest occurs after 10 passages in the absence of hTERT immortalization. (**C**) Cluster dendrogram showing the construction of replication module fingerprints by estimating the adjacencies of the input CpGs (hTERT training + liver aging), which was determined by extracting the highest loading CpGs in a replication trained PC model on all array CpGs. (**D**) Summary table displaying Pearson age correlation of all PC clock module measures. Note that the correlation from the hTERT dataset used during training was excluded from the hTERT validation samples. In addition, liver, developing brain, adult brain, blood, and skin were assessed as age correlations; fetal astrocytes and primary astrocytes were assessed as cPD correlations; and dermal and mammary cell lines were assessed as passage correlations due to limited cPD data information. * = the modules (yellow/tan) that were considered the most physiologically relevant and thus were used in subsequent analysis.

Human telomerase reverse transcriptase (hTERT)–immortalized fetal astrocytes were serially passaged, and DNAm was assessed longitudinally at each passage (Fig. 1A). We selected immortalized human fetal astrocytes as our model of choice as we reasoned that fetal cells would exhibit less fitness selection upon culturing, in comparison to adult primary cells. We also hypothesized that replication-driven DNAm changes would be cell type independent. In addition, immortalized astrocytes are commonly used as glial disease models due to their similarities in signaling to primary astrocytes and dramatically improved proliferation in culture, providing a physiologically relevant model with extended proliferative potential (*25*, *26*). The cellular life span of the immortalized astrocytes in our study was extended by more than 700% compared to nonimmortalized astrocytes. After 73+ cumulative population doublings (cPDs; when we ended data collection), cells showed no sign of growth arrest, genomic instability, or telomere erosion, allowing us to better isolate the effect of replication-based epigenetic changes (Fig. 1B and fig. S1).

As a first step toward identifying a signature of cell division, we conducted consensus network analysis to identify modules of comethylated CpGs (*27*). In brief, a subset of 20,101 CpGs were initially selected on the basis of their normalized loading scores from elastic net selected principal components (PCs) that tracked with cPD in our in vitro model (fig. S2). Clustering of these CpGs was then carried out on the basis of consensus clustering between our in vitro model and multi-age samples from human liver (*N* = 85, 23 to 83 years old). This enabled us to segregate physiologically relevant replication signals from cell culture artifacts (Fig. 1C). It also provided a physiological model while still limiting training to one tissue (liver).

The association of 14 consensus modules with number of cell divisions (cPDs) in vitro or aging in vivo was assessed across a diverse spectrum of cells and tissues. Two modules—termed “yellow” and “tan”— stand out as having signatures that consistently increased with proliferation and aging (Fig. 1D and figs. S3 and S4). The yellow and tan modules are the most physiologically relevant replication fingerprints, with strong correlations with age across a variety of in vivo tissues, including liver (*r*_yellow_ = 0.70, *r*_tan_ = 0.58, *N* = 85, 23 to 83 years old), skin (*r*_yellow_ = 0.66, *r*_tan_ = 0.59, *N* = 91, 20 to 90 years old), developing brain (*r*_yellow_ = 0.78, *r*_tan_ = 0.83, *N* = 173, 0 to 18 years old), and adult brain (*r*_yellow_ = 0.57, *r*_tan_ = 0.66, *N* = 502, 18 to 97 years old), and blood (*r*_yellow_ = 0.39, *r*_tan_ = 0.14, *N* = 2478, 40 to 92 years old) (Fig. 1D and fig. S3C). They also tracked serial passaging of primary cultures of fetal astrocytes (*r*_yellow_ = 0.93, *r*_tan_ = 0.88), primary astrocytes (*r*_yellow_ = 0.43, *r*_tan_ = 0.34), dermal fibroblasts (*r*_yellow_ = 0.93, *r*_tan_ = 0.8), and mammary fibroblasts (*r*_yellow_ = 0.47, *r*_tan_ = 0.79) (Fig. 1D). For these reasons, the CpGs in the yellow and tan modules served as the basis for all other downstream analyses in the study. Further details on the training, validation, and selection of the CpG modules are found in the computational section of Materials and Methods and the Supplementary Materials (figs. S4 and S5). Module occupancy of all module CpG sites can be found in table S1.

**Fig. 2.**
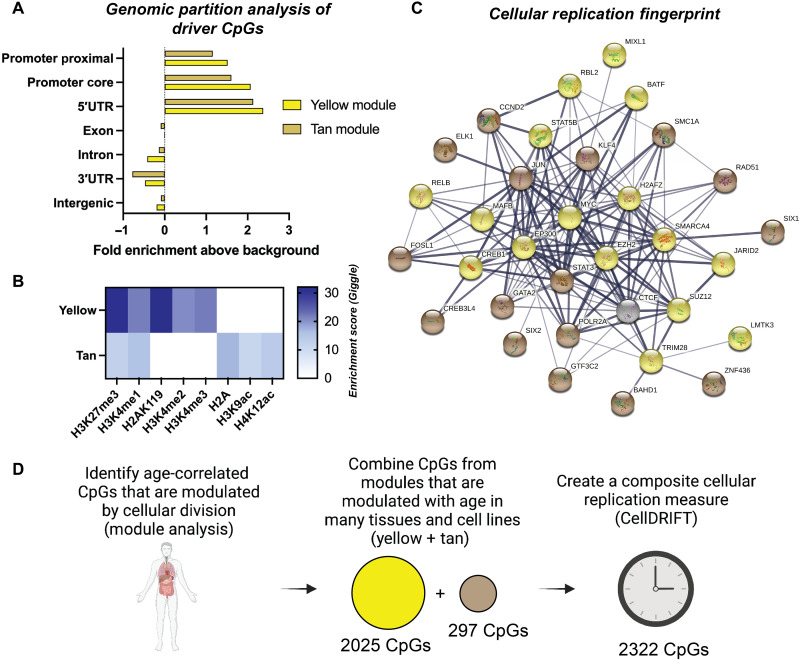
Replication-driven aging signals are enriched in PcG factor 2 domains and cell cycle regulators. (**A**) Genomic partition distribution plot showing that yellow and tan module driver CpGs are enriched in promoter (proximal and core) and 5′UTR regions compared to background. (**B**) Heatmap demonstrating histone enrichment score of module CpGs from Cistrome genome enrichment analysis. (**C**) String analysis of TFs and chromatin regulators enriched in CpGs from yellow and tan modules. Note that genomic partition, histone enrichment (B), and TF/chromatin regulator fingerprint analysis (C) were determined from conducting partition/Cistrome genome enrichment analysis on the top 100 kME CpGs from each module. The top 20 enriched genes were analyzed in the string analysis (C), with weakly connected nodes excluded. Final network strength and connectivity are based on line thickness, and color grouping is based on module occupancy, with yellow = yellow module, tan = tan module, and gray = both modules. (**D**) Schematic displaying selection process of isolating CpGs that are established by repeated cellular divisions and are also modulated with age in many tissues (yellow + tan module CpGs). The composite CpGs were used to create the measured CellDRIFT, which is used in subsequent analysis.

The most interconnected yellow and tan module CpGs (determined via eigengene-based connectivities (kME) are enriched in promoter core and proximal and 5′ untranslated region (5′UTR) regions (Fig. 2A and fig. S2). In addition, these replication fingerprint CpGs are enriched in polycomb repressive complex 2 (PRC2), pluripotency factors, and cell cycle regulator domains. Enrichment was assessed using the Cistrome database, which tests overlap for specific genomic locations of interest based on biorepository data from The Encyclopedia of DNA Elements (ENCODE) and includes information on specific histone marks, transcription factors (TFs), and chromatin regulators (fig. S6). Both modules were enriched in regions marked by histone 3 lysine 27 trimethylation (H3K27me3), which included both somatic and stem cell datasets (Fig. 2B). When testing for overlap with known TF binding sites, we observed enrichment in the yellow module with TFs that formed an interactive STRING protein network with PRC2 domains, such as the catalytic subunit enhancer of zeste homolog 2 (EZH2) and interacting cofactors polycomb protein SUZ12 (SUZ12), histone acetyltransferase p300 (EP300), jumonji AT rich interactive domain (JARID2), and tripartite motif-containing 28 (TRIM28) (Fig. 2C). PRC2 elements were also some of the most enriched by absolute score (fig. S6). This is of particular interest since PRC2 domains are implicated in the development and maintenance of many cancer types (*28*). PRC2 is a trimeric multiprotein complex (EZH2/EED/SUZ12), although it interacts with many other upstream and downstream cofactors like EP300, JARID2, and TRIM28, all of which have profound impacts on controlling cellular differentiation, signaling, and genome-wide regulation (*29*). Our enrichment data suggest that the epigenome may contribute to replication-driven dysregulation through many PRC2 component interactions, including both upstream and downstream regulators in addition to the trimeric core. CpGs in the tan module exhibited enrichment for additional noteworthy TFs, including kruppel-like factor 4 (KLF4), DNA repair protein RAD51 (RAD51), and signal transducer and activator of transcription 3 (STAT3) (Fig. 2C). KLF4 is a pluripotency factor and considered to be a tumor suppressor in many types of cancer, while RAD51 and STAT3 may predispose cells to cancer through faulty DNA repair or signal activation (*30*–*37*). The yellow and tan module TF-enriched hits were distinct, with the only overlap being CCCTC-binding factor (CTCF), suggesting that they represent two independent replication signals.

### Evaluation of replication fingerprint (CellDRIFT) in cancer patients, high–cancer risk individuals, and tissues with varying replicative history

To directly test the associations between these signals and changes in aging and cancer, we created a composite measure from the 2322 CpGs in the yellow and tan modules, termed CellDRIFT (Cellular Division and Replication Induced FingerprinT) (Fig. 2D and fig. S7).

**Fig. 3.**
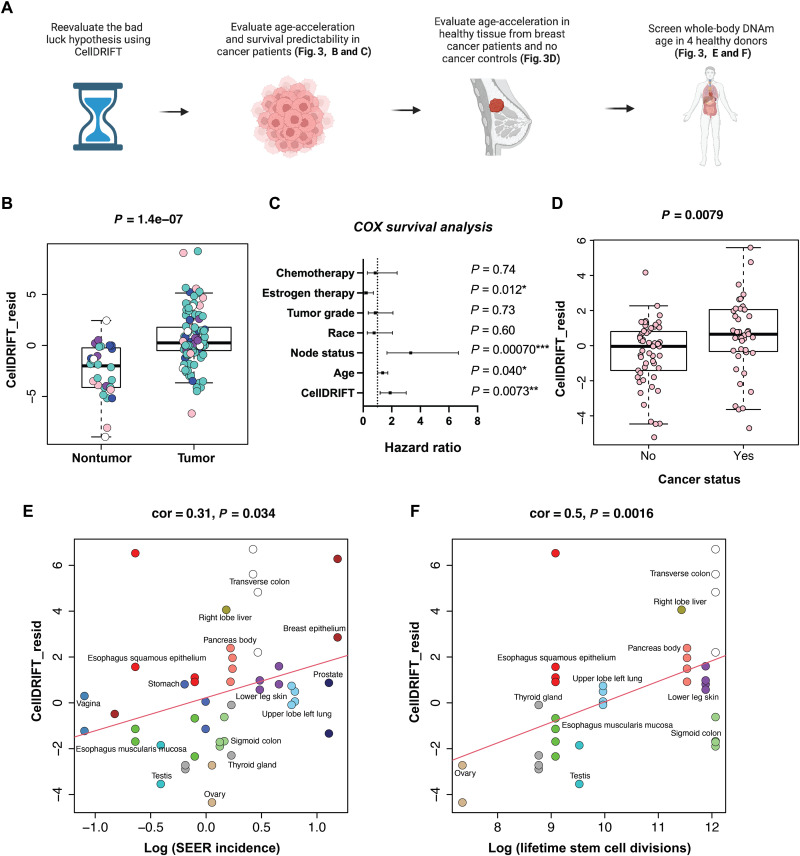
CellDRIFT predicts accelerated epigenetic risk in cancer tissue and healthy tissue of breast cancer patients and is correlated with cancer risk and lifetime stem cell divisions in whole-body tissue analysis. (**A**) Schematic displaying process for determining whether CellDRIFT captures propensity of tumorigenesis. (**B**) Pooled cancer and normal tissue from breast, colon, lung, pancreas, and thyroid cancer patients and controls, evaluated via CellDRIFT for DNAm acceleration in cancer tissue (GSE53051). Teal, thyroid; pink, breast; white, lung; purple, pancreas; and blue, colon. DNAmAge scores were residualized by age, sex, and tissue type. (**C**) Cox survival analysis of breast cancer patients from GSE37754, demonstrating that CellDRIFT is predictive of patient survival. Note that the hazard ratio for age was calculated as a risk increase per 10 years of life, and CellDRIFT was standardized by SD. (**D**) Differences in CellDRIFT epigenetic risk in healthy breast tissue of known breast cancer patients and participants with no history of prior breast cancer. DNAmAge scores were residualized by age, menopause status, and BMI before analysis. (**E**) Whole-body DNAmAge analysis from CellDRIFT in relation to propensity for cancer (SEER incidence per 100,000 people) of 14 different tissues from 4 individuals (ENTEx study). (**F**) Plot showing correlation between lifetime stem cell divisions and DNAmAge from CellDRIFT in nonzero cancer risk tissues from (E). Only tissues with reported lifetime stem cell division from Tomasetti and Vogelstein (*4*) were analyzed.

Malignant cells outgrow healthy cells via a number of proliferative mechanisms, including reduced activity of tumor suppressor genes, increased expression of oncogenes, chromatin dysregulation, and altered transcription (*19*, *38*). Many of these features, particularly epigenetic remodeling, may be acquired progressively over time before transformation. Unlike the bad luck and/or two-hit hypotheses, which describe mutations or a “hit” as the cancer-prone tipping point, the gradual and subversive epigenetic remodeling that likely is occurring as cells “tick” throughout life provides a path for understanding aging and cancer risk years before phenotypic penetrance. While our measure was not trained on cancer in any way, we hypothesize that CellDRIFT will capture aspects of premalignant changes as they accumulate and we predict that CellDRIFT (i) increases in tumors compared to normal tissues, (ii) will be higher in normal tissues from individuals who develop cancer versus healthy controls, and (iii) will be higher in tissues with greater proliferative activity and subsequent cancer risk (Fig. 3A).

To test this, we analyzed CellDRIFT in cancers of the thyroid, breast, lung, pancreas, and colon. We observed a significant increase in CellDRIFT (adjusted for age, sex, and tissue type) among the pooled tumor samples in comparison to corresponding normal tissues (*P* = 1.4 × 10^−0.7^) (Fig. 3B). Stratified analysis was also performed by cancer subtype. All tissues showed expected directionality; however, many were underpowered and only thyroid (*P* = 3.8 × 10^−0.5^) and breast (*P* = 0.021) cancers reached statistical significance (fig. S8). In moving forward, it will be informative to further explore associations across various cancer subtypes in larger studies. We followed up by analyzing a larger breast cancer cohort with reported survival data and similarly found that CellDRIFT from tumors is predictive of overall survival, even after adjusting for age, treatment, race, tumor grade (clinical staging), and node status (hazard ratio = 1.89, *P* = 0.0073) (Fig. 3C). This suggests that the epigenetic changes modeled by CellDRIFT capture cancer aggression and fitness and may even be useful for cancer pathologists in the future as a secondary diagnostics and prognostic end point. To provide such a tool, we created a GitHub R package for quick and easy calculation of CellDRIFT. Before any clinical implementation, additional validation is needed, although the package is uniquely suited to serve as a resource in the laboratory to explore underlying factors that may contribute to poor prognosis. Careful assessment of culture methods for cells used in clinical context is also important; thus, CellDRIFT may be used to screen out existing cellular stocks if passage information is unknown.

The next question, which we proposed would be the most important, but also the most difficult to detect, was determining whether CellDRIFT could predict accelerated drift—or high-risk patients—in pre-diseased healthy tissues. To accomplish this, we evaluated CellDRIFT in normal breast tissue from patients with and without diagnosis of breast cancer (before treatment) (*39*), hypothesizing that individuals who develop cancer in a particular tissue may do so as a result of more advanced and deleterious epigenetic modifications in the normal aging tissue before tumorigenesis—as captured by CellDRIFT (Fig. 3D). As hypothesized, we find that CellDRIFT is elevated in normal tissue from breast cancer patients compared to individuals who never had breast cancer, even after residualizing for age, menopause status, and body mass index (BMI) (*P* = 0.0079; Fig. 3D). Our results suggest that individual aging differences may precede and even promote the likelihood of a stochastic occurrence of a “bad luck” event that drives cancer formation. Additional longitudinal biopsy datasets will help clarify this conclusion.

Last, in accordance with the findings from Tomasetti and Vogelstein (*4*) and others (*24*), we reasoned that not all tissues would display the same replicative DNAm signatures and that cancer susceptibility (lifetime tissue-specific cancer risk) and tissue-specific stem cell division rates (replicative activity) would correlate with the degree of epigenetic changes captured by CellDRIFT in various tissues. To test this, we estimated CellDRIFT in samples from ENTEx, which profiled 29 tissues from four patient donors (*40*). For our primary analysis, we restricted tissues to those with reported lifetime cancer risk [according to the National Cancer Institute’s Surveillance, Epidemiology, and End Results (SEER) program]. Our results showed that CellDRIFT was positively correlated with both tissue-specific cancer risk (cor = 0.31, *P* = 0.034) (Fig. 3E) and lifetime stem cell divisions (cor = 0.5, *P* = 0.0016) (Fig. 3F). Overall, this suggests that more proliferative tissues may have greater CellDRIFT, and this may explain the higher propensity for cancer over the lifetime of that tissue. The stem cell division prediction traversed nearly six orders of magnitude (i.e., ovary versus transverse colon), demonstrating the tight link between replicative activity and epigenetic regulation. In addition, even when near-zero cancer risk tissues, like the ascending aorta and gastrocnemius medialis, were included as a sensitivity analysis, the association holds (cor = 0.3, *P* = 0.0031) (fig. S9), suggesting that low-cancer risk tissues exhibit less epigenetic drift potentially because of their low replicative activity. In short, CellDRIFT provides a tool for future studies of replication-associated epigenetic mechanisms that may underlie Tomasetti and Vogelstein’s initial observation and provides further experimental evidence that links cellular replication to differential cancer risk and aging.

### Assessment of OCT4, SOX2, KLF4, and MYC (OSKM) reprogramming and long-term passaging in pluripotent stem cells

**Fig. 4.**
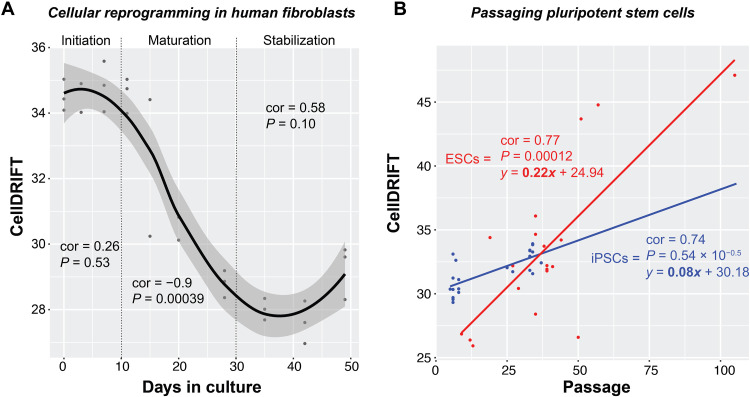
CellDRIFT is turned back upon reprogramming in human fibroblasts, but is not stabilized with additional passages. (**A**) Scatterplot showing reprogramming trajectory in CellDRIFT from OSKM reprogrammed human dermal fibroblasts from GSE54848, analyzed separately for initiation, maturation, and stabilization phases. (**B**) Scatterplot demonstrating that passaging increases CellDRIFT in pooled iPSCs reprogrammed from human dermal fibroblast’s via OSKM and ESCs. Note that 11 different iPSC lines were included, which were generated from 11 clones, and 15 ESC lines were included (GSE31848). Correlation and statistical significance were determined via Pearson correlations in each phase and in the extended passaging plot.

Our final question was to determine whether cellular reprogramming (*37*) can reset or modulate CellDRIFT. We analyzed time-course data from human fibroblasts reprogrammed to induced pluripotent stem cells (iPSCs) and tracked CellDRIFT throughout the three phases of Yamanaka factor reprogramming: initiation, maturation, and stabilization. No change was seen during the early initiation phase of reprogramming. However, we observed a dramatic decrease in the CellDRIFT signal during the maturation phase, which coincides with dedifferentiation or transition to pluripotency (cor = −0.9, *P* = 0.00039) (Fig. 4A). Upon passaging in the stabilization phase, we observed a slight “uptick” in CellDRIFT, although it did not reach statistical significance, potentially due to a lack of statistical power (cor = 0.58, *P* = 0.10) (Fig. 4A). For this reason, we analyzed an additional iPSC and embryonic stem cell (ESC) dataset that reported extended passaging (Fig. 4B). In both the dermal fibroblast–derived iPSC and ESC lines, passaging strongly induced further CellDRIFT (cor = 0.74, *P* = 5.4 × 10^−5^ and cor = 0.77, *P* = 0.00012, respectively), suggesting that pluripotent cells, despite having long-term passaging abilities (*41*), are not immune to replication-related epigenetic drift (Fig. 4B). We also analyzed a second ESC dataset (*42*) that reported multiple passaging conditions (enzyme versus mechanical passaging and mouse feeder versus extracellular matrix) and found that drift is sustained even out to passage 161 (fig. S10).

Our study defines a signature of epigenetic remodeling observed in vitro as a “pure” function of DNA replication. We show that this signature increases as a function of age in vivo and provides evidence to suggest that it may underlie an age-related transition of tissues from a (youthful) state of normal tissue and cell functioning toward an (aged) tipping point of tumorigenic transformation. The epigenetic changes captured in our model are consistent with prior characterizations in aging and cancer, implicating EZH2 binding sites (*43*) and chromatin accessibility hotspots (*44*). The key takeaway is that by using a signature of molecular changes arising via replication (presented here as CellDRIFT), it is possible to quantify and track the inevitable entropic disorder that occurs with aging. While tissue-specific stem cell division rates may set a baseline risk of cancer development in various tissues, individual differences in cancer risk may not be entirely luck based and instead might in part reflect differential epigenetic aging rates. Hence, there is a need for research into interventions to slow or reverse the accumulation of epigenetic changes with age. While applications like epigenetic reprogramming demonstrate that modulation is possible, issues surrounding dedifferentiation of cells, as well as feasibility and targeted efficiency mean that such techniques are long from practical applications. This begs the question of whether positive lifestyle factors may modulate our luck beyond chance.

## MATERIALS AND METHODS

### Experimental

#### Fetal astrocyte extraction

Four fetal-mortal astrocyte cell lines were derived from the cerebral cortex of two different donors (ScienCell, #1800): donor 1, which was split into −hTERT1, −hTERT2, and −hTERT3 cell lines and was used in training experiments, and donor 2, which was split into −hTERT4. Tissue was received by ScienCell Research Laboratories from nonprofit tissue providers, obtained with informed consent of donor’s family aged over 18 years and under established protocols in compliance with an institutional review board and local, state, and federal laws. No payment, commercial rights, or financial rights were provided to the donor family. Further details can be obtained from ScienCell Research Laboratories.

#### Primary astrocyte extraction

Three primary human astrocyte cell lines were derived from the cerebral cortex of one 21-year-old male donor (Creative Biolabs, #NCL-2103-P104). The 21M donor was split into Astro1, Astro2, and Astro3 cell lines, which were then subsequently exhaustively passaged. All tissue collection procedures used by Creative Biolabs, and its partners, were performed in compliance with institutional review boards and local, state, and federal laws. Further details, including specific informed consent practices, can be obtained by contacting Creative Biolabs directly.

#### Immortalized (hTERT) astrocyte preparation

hTERT immortalized fetal astrocyte cell lines were supplied by Applied Biological Materials (Abm, #T0281). The immortalized cells were supplied at passage 12, with passage reporting starting after hTERT immortalization. Upon receiving, we subsequently split the immortalized fetal donor into three hTERT cell lines, +hTERT1, +hTERT2, and +hTERT3, which were then passaged to p27. Further details about plasmids used in transfection and details about donor sourcing can be obtained from Abm.

#### Astrocyte replicative passaging and cellular culturing

Fetal cells were exhaustively passaged and split a total of 10 times (9 to 15 cPDs, depending on replicate), where β-galactosidase (β-gal) activity (C12FDG) was measured using flow cytometry or confocal microscopy at each passage to confirm that exhaustive replication was achieved. Primary astrocytes were also exhaustively passaged and split a total of 10 times (eight to nine cPDs, depending on replicate). hTERT immortalized astrocytes were passaged a total of 27 times (73 to 75 cPDs, depending on replicate).

All cell lines were seeded at 8000 cells/cm^2^ (0.5 × 10^6^ cells per p100) with appropriate growth media and supplements [complete astrocyte medium containing amino acids, vitamins, hormones, trace minerals, 2% fetal bovine serum, and 1% penicillin-streptomycin in Hepes (pH 7.4) bicarbonate buffer; ScienCell, #1801] to promote cell adhesion and growth. Of note, poly-l-lysine was not required for adequate cell adhesion. All cells were grown under normoxic conditions (20% O_2_, 5% CO_2_) at 37°C. Cells were split [0.05% trypsin-EDTA (Gibco, #25300-54)] when they reached approximately 90% confluence or when static growth was achieved. Cells were counted using the Invitrogen countess and cell counting chamber slide with trypan blue. cPD was calculated using the initial and final cell density, as determined by the countess (2*^x^* = FD/ID, where *x* is the population doubling, FD is the final cell density, and ID is the initial cell density).

#### DNA preparation

Longitudinal samples were collected at every passage, and DNA was extracted using the Qiagen DNeasy Blood and Tissue Kit (#69504). Note that samples were treated with proteinase K and ribonuclease A and eluted with 200 μl of elution buffer. Following final elution, DNA was verified using NanoDrop (Thermo Fisher Scientific) and Qubit fluorometer (Invitrogen). Spin concentration was used as necessary with low DNA content samples. Before library preparation, we used a Qubit fluorometer (Invitrogen) to quantify the extracted genomic DNA.

#### β*-Gal confocal microscopy*

To assess senescence status, we used a β-gal imaging method. In brief, cells were split into 12-well dishes (0.125 × 10^6^ cells per well) with a glass cover slide at the bottom of each well and allowed to settle for 24 hours. Cells were first pretreated with bafilomycin A1 (Selleckchem, S1413, 622.83 g/mol, 100 μM stock). Existing medium was aspirated, and then cells were washed with phosphate-buffered saline (PBS) and replaced with treated bafilomycin A1 media for 30 min at a final concentration of 100 nM. Following bafilomycin A1 pretreatment to normalize lysosome activity, C12FDG (Invitrogen, #D2893, 853.92 g/mol, 10 mM stock) was added directly to the existing media for 90 min at a final concentration of 10 μM. Note that because of light sensitivity, exchange was conducted in a dark environment. Following bafilomycin A1 and C12FDG treatments, medium was aspirated, and cells were washed with PBS three times, fixed with 4% paraformaldehyde/PBS (10 min), followed by two PBS washes, and then counterstained with 4′,6-diamidino-2-phenylindole (DAPI; Invitrogen, #P36935) and mounted onto coverslips. Fixed cells were immediately imaged using a ZOE fluorescent cell imager (Bio-Rad). Percent cell positively was calculated using ImageJ with a background/negative threshold value. Any cells above this threshold were considered positive. All cells in each image frame were counted.

#### F-actin (Phalloidin CruzFluor 532) confocal microscopy

Passage 3, 6, and 10 fetal astrocytes (−hTERT1 to −hTERT3) were concurrently passaged to dynamically visualize changes in cellular morphology. In brief, cells were split into 12-well dishes (0.125 × 10^6^ cells per well) with a glass cover slide at the bottom of each well. Cells were allowed to grow for 5 days before assessment. Following growth, cells were fixed using 4% formaldehyde in PBS for 30 min. In summary, loose cells and media were aspirated, washed two times with PBS, and then fixed with 4% formaldehyde for 30 min. After fixation, cells were washed two times with PBS, and then Phalloidin CruzFluor 532 (1×) (Santa Cruz Biotechnology, #363793) conjugate was added for 90 min. Following conjugation, cells were gently rinsed twice with PBS and then twice with H_2_0. Last, cells were counterstained with DAPI (Invitrogen, #P36935), mounted onto coverslips, and imaged with a ZOE fluorescent cell imager (Bio-Rad).

#### Absolute telomere length quantitative polymerase chain reaction quantification

Telomere attrition was calculated as a percent change from baseline using absolute telomere length assessed using a quantitative polymerase chain reaction (qPCR) kit from ScienCell (#8918). −hTERT1 and hTERT1 cell lines were used in the assessment. More specifically, −hTERT cells passaged 2, 4, 6, 8, and 10 times and hTERT1 cells passaged 13, 15, 17, 19, 21, 23, 25, and 27 times were used in the final telomere attrition reporting and comparisons.

### Computational

#### Data processing

All samples were assigned a single-blinded code and randomized for library preparation and sequencing to control for any batch errors. DNAm data were generated using the Infinium HumanMethylation850 BeadChip, preprocessed using minfi (*45*), and normalized using the noob method (*46*). Before analysis, all sex chromosome CpGs were excluded, and for training and validation purposes, we aligned the CpGs to the 450k array, giving a final matrix of 442,242 CpGs.

#### Training and validation of DNAmImmort, module clocks, CellDRIFT, and PC clocks

R was the primary platform used throughout the study (version 4.1.1). Prism (version 9) was also used for certain statistical analysis and plotting. DNAmImmort was constructed using only passaged hTERT cells. In brief, three hTERT cell lines were used in the training and validation process, all from the same donor and immortalization pair. +hTERT1 and +hTERT2 (*n* = 31, p13 to p27) were used in training, hereafter referred to as hTERT training, and +hTERT3 (*n* = 14, p13 to p27) was used in validation, hereafter referred to as hTERT validation. In summary, principal components analysis (PCA) was conducted on the hTERT training samples, and then we used elastic net regression modeling to select the final 16 PCs used in the measure based on using cPD as the training variable for calibration. The 16 PCs were composed of PCloading scores for all 442,242 CpGs, with elastic net coefficients based on lambda penalty values representing the lowest mean-squared error, selected via 10-fold cross validation.

From modules identified according to Weighted gene co-expression network analysis (WGCNA) in the following section, we trained a number of smaller clocks. These were trained using the same hTERT training and validation samples, except that PCA was conducted on the module CpGs, resulting in a measure with substantially reduced CpGs (turquoise, 4008 CpGs; blue, 3859 CpGs; brown, 2641 CpGs; yellow, 2025 CpGs; green, 1525 CpGs; red, 1252 CpGs; black, 912 CpGs; pink, 882 CpGs; magenta, 880 CpGs; purple, 756 CpGs; green-yellow, 721 CpGs; tan = 297 CpGs; salmon, 234 CpGs; and cyan, 108 CpGs). See more details below on the process of isolating the most physiologically relevant module CpGs.

To determine the cell and tissue age correlations, we first conducted PCA in each module CpG cohort (eigengene) in our in vitro replication data and then calculated the resulting variance (PC1) within each validation dataset (fig. S4B). Next, we trained elastic net selected PC clocks (DNAmModuleColor) as replication predictors in our in vitro model and then calculated DNAmAge Pearson correlations within each module clock and validation dataset (Fig. 1D). In both eigengene (PC1) and module clock analysis, the yellow and tan module CpGs were the most physiologically relevant replication fingerprints, with strong correlations across both in vivo tissues—liver, skin, developing and adult brain, and blood—and in vitro cell lines—fetal astrocytes, primary astrocytes, primary dermal fibroblasts, and primary mammary fibroblasts (Fig. 1D). In moving forward, the CpGs in these two modules served as the input CpGs for the final composite measure, CellDRIFT.

The final cellular replication measure—CellDRIFT—was trained from the combined CpGs from the yellow (2025) and tan (297) modules, which were selected from extensive in vitro and in vivo validation analysis to determine multitissue and physiological relevance (Fig. 1D). Instead of recalculating PCs for the new 2322 CpG cohort, we combined the independent PCs (62 total, 31 from each module) and conducted elastic net to select for the final PCs for inclusion in the final measure. This way, each signal had equal weight and possibility for contributing to the final measure and CpG occupancy was not a biasing factor. The following PCs were selected from each module: yellow (PC1, PC2, PC3, PC10, and PC17) and tan (PC1, PC2, PC3, PC4, PC5, PC7, PC10, and PC11). Further details on PC-trained measures can be found in our previous reports (*7*, *13*). We also constructed a GitHub package for easy calculation of CellDRIFT.

Validation of all measures was done first by assessing the DNAmAge of the hTERT validation data (*n* = 14), followed by various multitissue and additional in vitro datasets (fig. S3). Pearson correlations were used to determine associations between all measures and validation data presented in Fig. 1D. Note that liver, developing brain, adult brain, blood, and skin were assessed as age correlations; hTERT, fetal astrocytes, and primary astrocytes were assessed as cPD correlations; and dermal and mammary cell lines were assessed as passage correlations due to limited cPD data information.

As controls, we evaluated the traditionally trained ex vivo clocks PCHorvath1 and PCPhenoAGE and found that CellDRIFT outperformed the multitissue measure PCHorvath1, and performed similarly to PCPhenoAGE, which is remarkable considering that PhenoAGE was trained as a health span predictor and is highly associated with mortality, while CellDRIFT is only trained from in vitro cell divisions (fig. S11). We associated the improved cancer detection power of PCPhenoAGE over PCHorvath1 to its tighter association with all-cause mortality. PC clock calculations for PCHorvath1 and PCPhenoAGE were conducted using the data analysis pipeline from Higgins-Chen *et al.* (*13*).

#### WGCNA module construction and hierarchical clustering

We conducted consensus WGCNA (*27*) and hierarchical clustering to produce distinct DNAmAge signals, as we previously reported (*7*). In brief, we used two input datasets (hTERT training and liver aging), with the remaining datasets (hTERT validation, blood, brain, skin, primary astrocyte, fetal astrocyte, dermal, and mammary fibroblasts) excluded for validation purposes. In total, we used 20,101 CpGs in the consensus analysis. The CpGs included were the top driver CpGs of DNAmImmort, which were selected from normalizing PC loading scores and selecting the top values (fig. S2F). In brief, adjacencies were estimated for each dataset, which is based on biweight midcorrelations. These adjacencies were then converted to topological overlap matrices (TOMs), where a minimum dissimilarity score was calculated for each CpG pair across the two TOMs. Hierarchical clustering was then conducted with the following parameters: deepSplit = 1, cutHeight = 0.95, minClusterSize = 50, and distance = 1-consensus TOM, method = average. The resulting network produced 14 modules. No further module cutting was performed, with all CpGs in the input analysis being assigned a module. Following module construction, we estimated PC1 for each module using the hTERT training data (fig. S4B) and then applied this score to all validation data to serve as a validation metric of module connectivity and similarity. Module clocks were then trained from the module CpGs and taken forward to determine differences in DNAmAge between modules. For further information on module development, refer to Minteer *et al.* (*7*).

#### Cistrome genome enrichment analysis

To better determine the functionality of the CpGs selected from each module, we used the Cistrome gene analysis tool kit (http://dbtoolkit.cistrome.org/) to determine enriched genes and histone marks in each CpG dataset. In brief, we plotted all TFs and chromatin regulators with significant overlap with the CpG modules and then plotted a heatmap of the top five genes (average Giggle score across all GSM_ID hits) from each module together to compare module to module enrichment (fig. S6). Note that the summary module enrichment analysis used the top 100 CpGs from each module, determined from the kME score. Enriched genes were normalized by selecting 100 background CpGs from the original 440k training dataset and correcting for each GSM_IDs Giggle score. Enrichment analysis is displaying the average Giggle score across all GSM_IDs, with the top 5 for each module plotted in fig. S6Q and then the top 20 were fed into String analysis in Fig. 2C. Note that histone mark analysis was conducted on the top 100 kME CpGs and corrected for background hits, with the final heatmap displaying the top five marks by average Giggle score (Fig. 2B). Giggle score is a rank of genome significance between genomic locations of query file and thousands of genome files from databases like ENCODE. For further information on normalization and plotting Giggle scores, refer to Minteer *et al.* (*7*).

#### Additional analysis pipelines

Genomic partitioning and CpG locations were determined using LolaWeb (http://lolaweb.databio.org/). STRING protein-protein network analysis was conducted using the STRING database (https://string-db.org/). Medium confidence (0.4) was set as the minimum interaction score threshold.

#### Statistical analysis and R packages

Plotting and module development were conducted using the WGCNA package. Additional plotting was done using the ggplot2 package. Elastic net modeling was conducted using the glmnet package. Survival analysis and Cox hazard ratio analysis were conducted using the survival, survminer, and dplyr packages. Hazard ratios were calculated in reference to survival data from a cohort of breast cancer patients (GSE37754), with the interaction variable being the CellDRIFT or PCclock scores. Note that the hazard ratio for age was calculated as a risk increase per 10 years of life, and CellDRIFT was standardized by SD. Additional packages of interest used in this study were BiocManager, lattice, viridis, RColorBrewer, reshape, and GEOquery. Pearson correlations were used to assess age and passage associations, the Kruskal-Wallis test was used in group-group and multigroup comparisons, and biweight midcorrelations were used for determining adjacencies in module construction analysis.

#### Data accessibility and usage

All data used in this study are summarized below: training: hTERT training data [this study (GSE226079), *n* = 31 samples]; validation: hTERT validation data [this study (GSE226079), *n* = 14], liver aging (GSE48325), brain aging (GSE74193), blood aging (GSE40279), skin aging (GSE52980), primary astrocytes [this study (GSE226079), *n* = 25], fetal astrocytes (this study/GSE202554), primary dermal fibroblast and primary mammary fibroblasts (E-MTAB-8327); cancer: pooled cancer samples (GSE53051), breast cancer survival data (GSE37754), healthy breast tissue—Rozenblit *et al.* 2022, whole-body tissue (ENTEx study); reprogramming: Yamanaka fibroblast reprogramming and passaging data (GSE54848), iPSC/ESC passaging data (GSE31848), and additional variable passaging conditions ESC dataset (GSE56851).

#### CellDRIFT GitHub package

We created a GitHub R package to help facilitate easy calculation of CellDRIFT in external datasets. You can install the CellDRIFT like so: devtools::install_github(“MorganLevineLab/CellDRIFT”). The GitHub package can also be accessed from the following link, where details can be explored: https://github.com/MorganLevineLab/CellDRIFT. Code and details can also be accessed via Zenodo (doi = 7693699, link = https://zenodo.org/record/7693699#.ZAEf5OzMKDU).
