# Supplementary material for: More than bad luck: Cancer and aging are linked to replication-driven changes to the epigenome
Source: Sci Adv. 2023 Jul 19;9(29):eadf4163. doi: 10.1126/sciadv.adf4163 (PMC10355820; doi:10.1126/sciadv.adf4163)
Supplement: Supplementary file 1 — Figs. S1 to S11 Legend for table S1 [file sciadv.adf4163_sm.pdf]

Science Advances
AAAS

Supplementary Materials for

More than bad luck: Cancer and aging are linked to replication-driven changes to the epigenome

Christopher J. Minteer *et al.*

Corresponding author: Morgan E. Levine, mlevine@altoslabs.com

Sci. Adv. **9**, eadf4163 (2023)
DOI: 10.1126/sciadv.adf4163

The PDF file includes:

Figs. S1 to S11
Legend for table S1

Other Supplementary Material for this manuscript includes the following:

Table S1

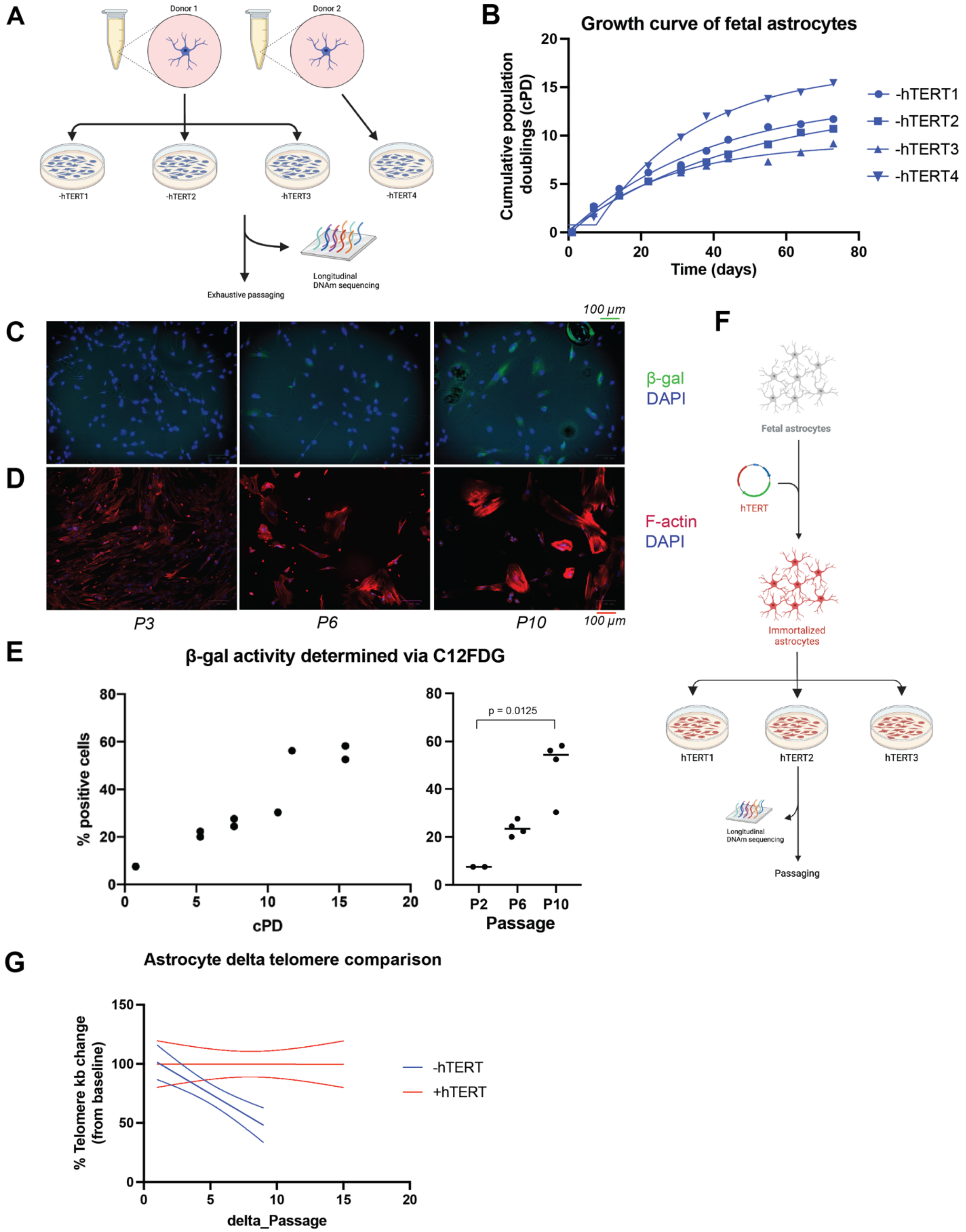
A
Donor 1
Donor 2
-hTERT1
-hTERT2
-hTERT3
-hTERT4
Exhaustive passaging
Longitudinal
DNAm sequencing
B
Growth curve of fetal astrocytes
Cumulative population doublings (cPD)
Time (days)
-hTERT1
-hTERT2
-hTERT3
-hTERT4
C
D
100 μm
β-gal
DAPI
F-actin
DAPI
P3
P6
P10
100 μm
E
β-gal activity determined via C12FDG
% positive cells
cPD
p = 0.0125
P2
P6
P10
Passage
F
Fetal astrocytes
hTERT
Immortalized
astrocytes
hTERT1
hTERT2
hTERT3
Longitudinal
DNAm sequencing
Passaging
G
Astrocyte delta telomere comparison
% Telomere kb change
(from baseline)
delta_Passage
-hTERT
+hTERT

Fig. S1: Astrocyte model development and passaging. (A) Schematic displaying method of creating the 4 mortal-fetal astrocyte cell lines (-hTERT1-4). 2 donors were used (Donor 1 = -hTERT1-3 and Donor 2 = -hTERT4). All replicates were then exhaustively passaged until senescence was achieved. **(B)** Plot displaying growth rate of mortal astrocytes, where growth arrest and senescence was achieved after 10x passages. **(C-D)** Representative confocal microscopy images of mortal-fetal astrocytes at P3, P6 and P10, displaying increase in senescence (β-gal) and enlarged cellular morphology (F-actin), counterstained against DAPI. **(E)** Image-J quantified β-gal activity of confocal microscopy images. **(F)** Schematic displaying method for creating the 3 immortalized-fetal astrocyte cell lines (+hTERT1-3). Note, 1 donor was used and following successful immortalization, the cells were split into 3 different cell lines (+hTERT1-3), which were then extensively passaged. Note, at the time of stopping the experiment the cells were P27, with no signs of growth arrest or senescence. Longitudinal DNAm from P13-P27 were used in subsequent PC clock creation of DNAmImmort, module clock creation and CellDRIFT. +hTERT1-2 were used in training and +hTERT3 was used as validation. **(G)** Absolute telomere length assessment of mortal (-hTERT) and immortalized (+hTERT) astrocytes demonstrating telomere erosion occurs in the absence of hTERT immortalization.

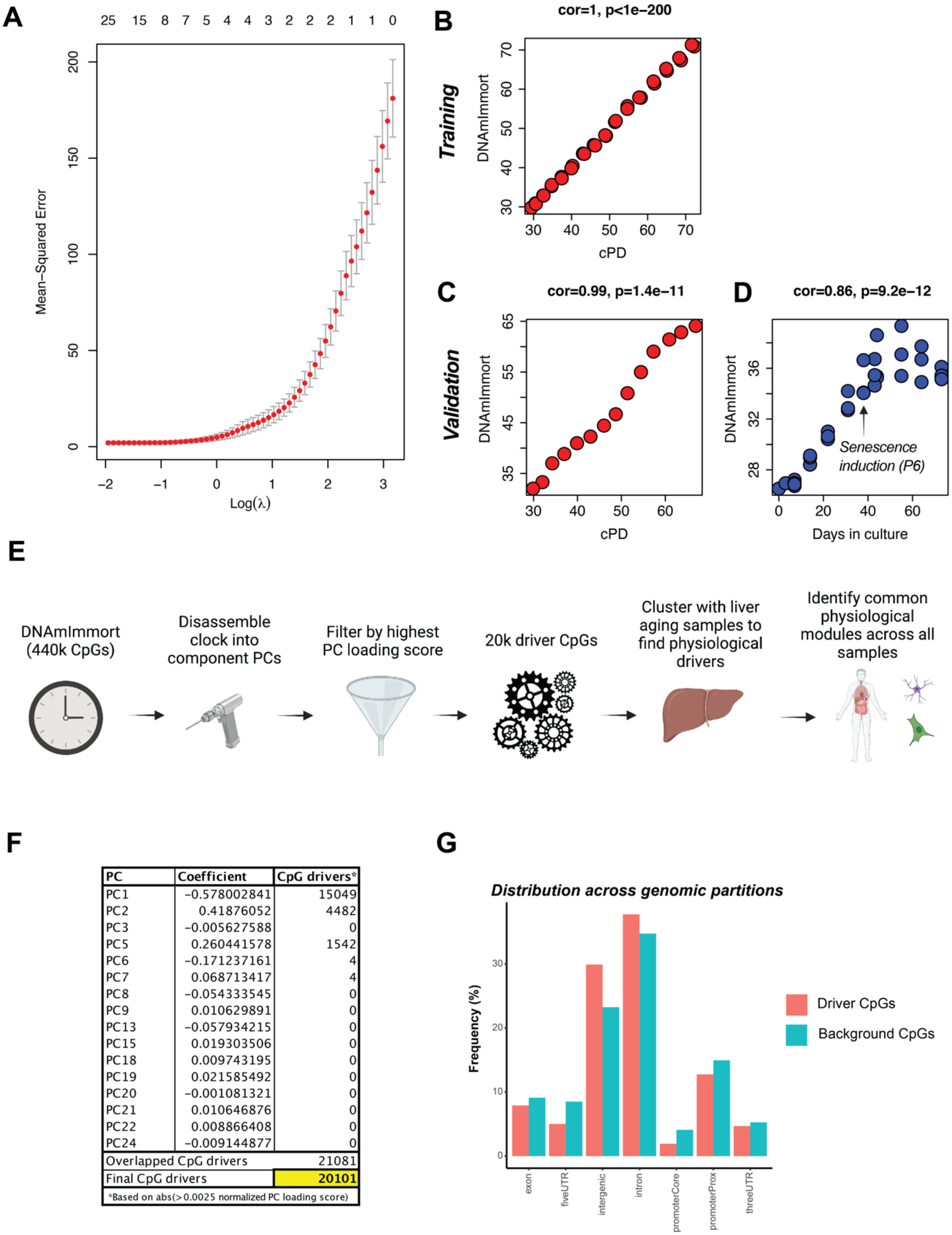

PC	Coefficient	CpG drivers*
PC1	−0.578002841	15049
PC2	0.41876052	4482
PC3	−0.005627588	0
PC5	0.260441578	1542
PC6	−0.171237161	4
PC7	0.068713417	4
PC8	−0.054333545	0
PC9	0.010629891	0
PC13	−0.057934215	0
PC15	0.019303506	0
PC18	0.009743195	0
PC19	0.021585492	0
PC20	−0.001081321	0
PC21	0.010646876	0
PC22	0.008866408	0
PC24	−0.009144877	0
Overlapped CpG drivers		21081
Final CpG drivers		20101

*Based on abs(>0.0025 normalized PC loading score)

H

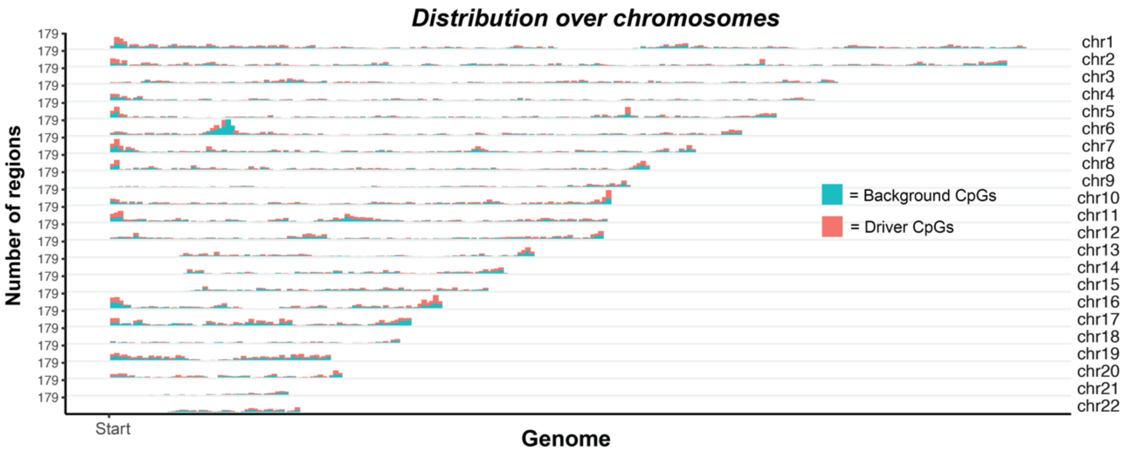
Distribution over chromosomes
Number of regions
179
chr1
chr2
chr3
chr4
chr5
chr6
chr7
chr8
chr9
chr10
chr11
chr12
chr13
chr14
chr15
chr16
chr17
chr18
chr19
chr20
chr21
chr22
= Background CpGs
= Driver CpGs
Start
Genome

I

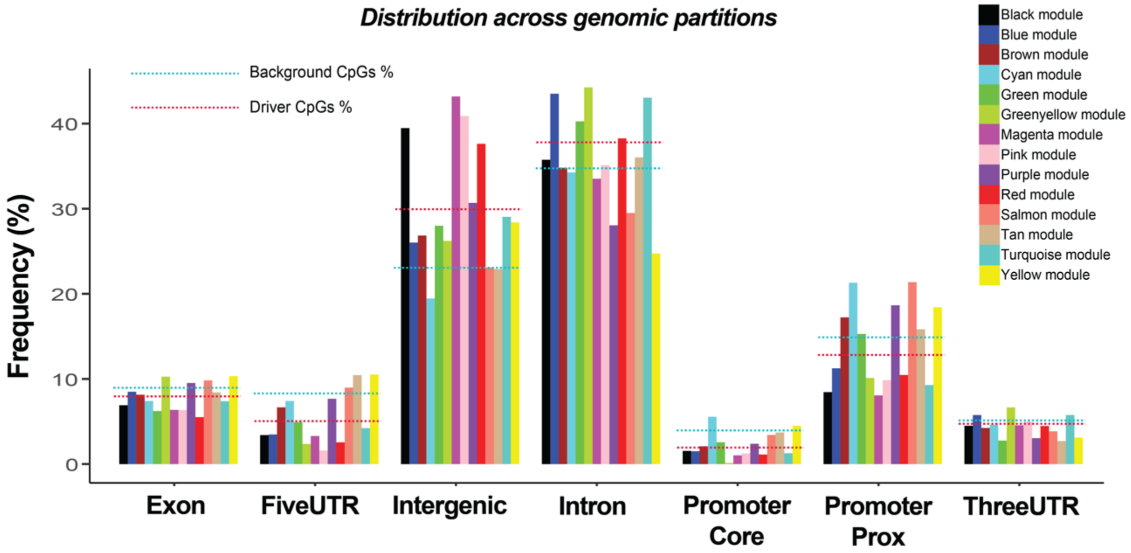
Distribution across genomic partitions
Background CpGs %
Driver CpGs %
Frequency (%)
0
10
20
30
40
Exon
FiveUTR
Intergenic
Intron
Promoter Core
Promoter Prox
ThreeUTR
Black module
Blue module
Brown module
Cyan module
Green module
Greenyellow module
Magenta module
Pink module
Purple module
Red module
Salmon module
Tan module
Turquoise module
Yellow module

J

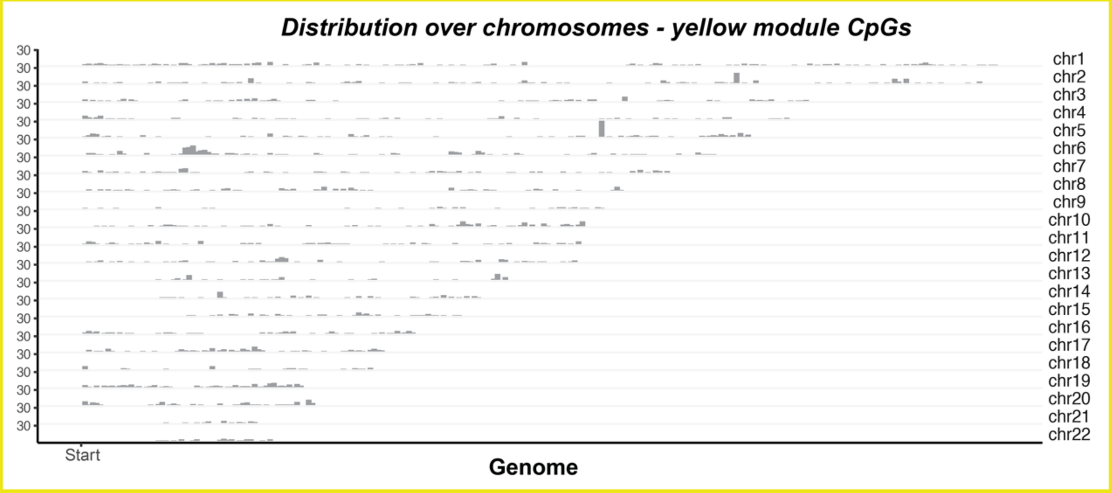
Distribution over chromosomes - yellow module CpGs
Number of regions
30
chr1
chr2
chr3
chr4
chr5
chr6
chr7
chr8
chr9
chr10
chr11
chr12
chr13
chr14
chr15
chr16
chr17
chr18
chr19
chr20
chr21
chr22
Start
Genome

K

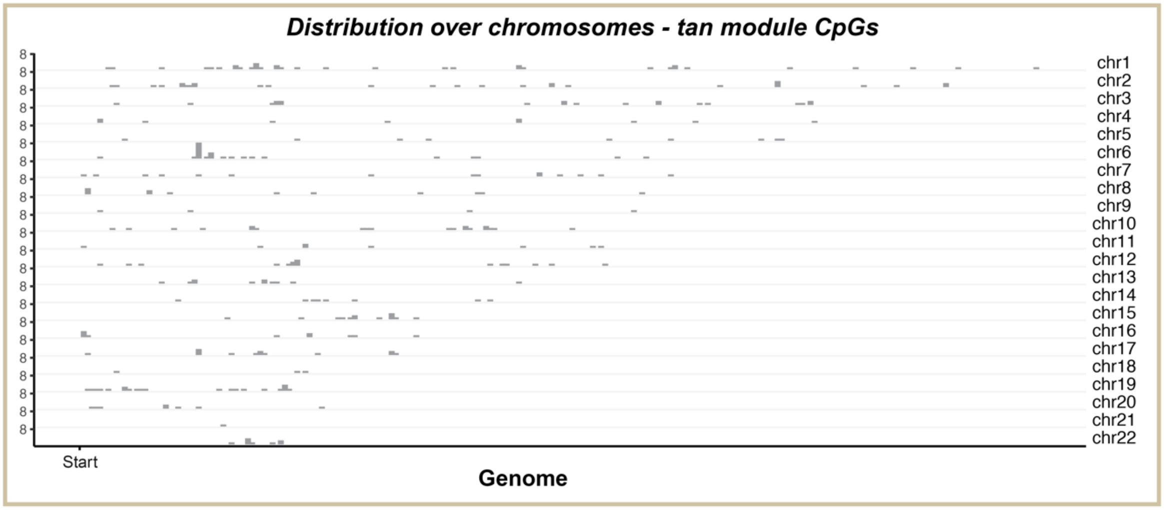
Distribution over chromosomes - tan module CpGs
Number of regions
Genome
Start
chr1
chr2
chr3
chr4
chr5
chr6
chr7
chr8
chr9
chr10
chr11
chr12
chr13
chr14
chr15
chr16
chr17
chr18
chr19
chr20
chr21
chr22

Fig. S2: Extracting cellular replication CpG drivers. (A) Elastic net lambda minimum selection of PCs for inclusion in final model, DNAmImmort. **(B)** hTERT PC clock – DNAmImmort – trained from hTERT1 and hTERT2 replicates and using cPD as input variable. **(C)** DNAmImmort validation data using hTERT3 replicate not used in model training and **(D)** assessment of DNAmImmort measure in mortal (-hTERT1-3) astrocytes, displaying reduced DNAm rate upon senescence induction. **(E)** Schematic displaying workflow of breaking the DNAmImmort measure into module drivers by conducting consensus clustering on the 20,101 CpG drivers, as determined by normalizing the PC loading scores. Note, hTERT1-2 replicates and liver aging samples were used in the clustering analysis to produce the 14 modules, then using the new module CpGs module clocks were trained using the same hTERT1-2 replicates and cPD as the input variable. **(F)** Summary table of all module components in PC clock, DNAmImmort, with extracted driver CpGs (20,101) selected by pulling CpGs with a normalized (absolute value of elastic net coefficient) PC loading score of >0.0025. In the selection analysis each PC was analyzed independently. **(G)** Genomic distribution plot displaying CpG locations/regions of driver and background CpGs, showing enrichment in intronic and intergenic regions of the DNAmImmort driver CpGs. **(H)** Chromosome distribution plot generated by LolaWeb displaying 20,101 driver CpGs vs. random 20,101 background CpGs from the original 440k sex-excluded CpGs of the original training dataset. **(I)** Genomic partition distribution across all module CpGs presented in Fig. 1C, plotted using LolaWeb. Dotted lines represent the genomic partition frequency of the 20,101 driver CpGs and randomly sampled 440k background CpGs. **(J)** Chromosome distribution plot generated by LolaWeb displaying yellow module CpGs. **(K)** Chromosome distribution plot generated by LolaWeb displaying tan module CpGs.

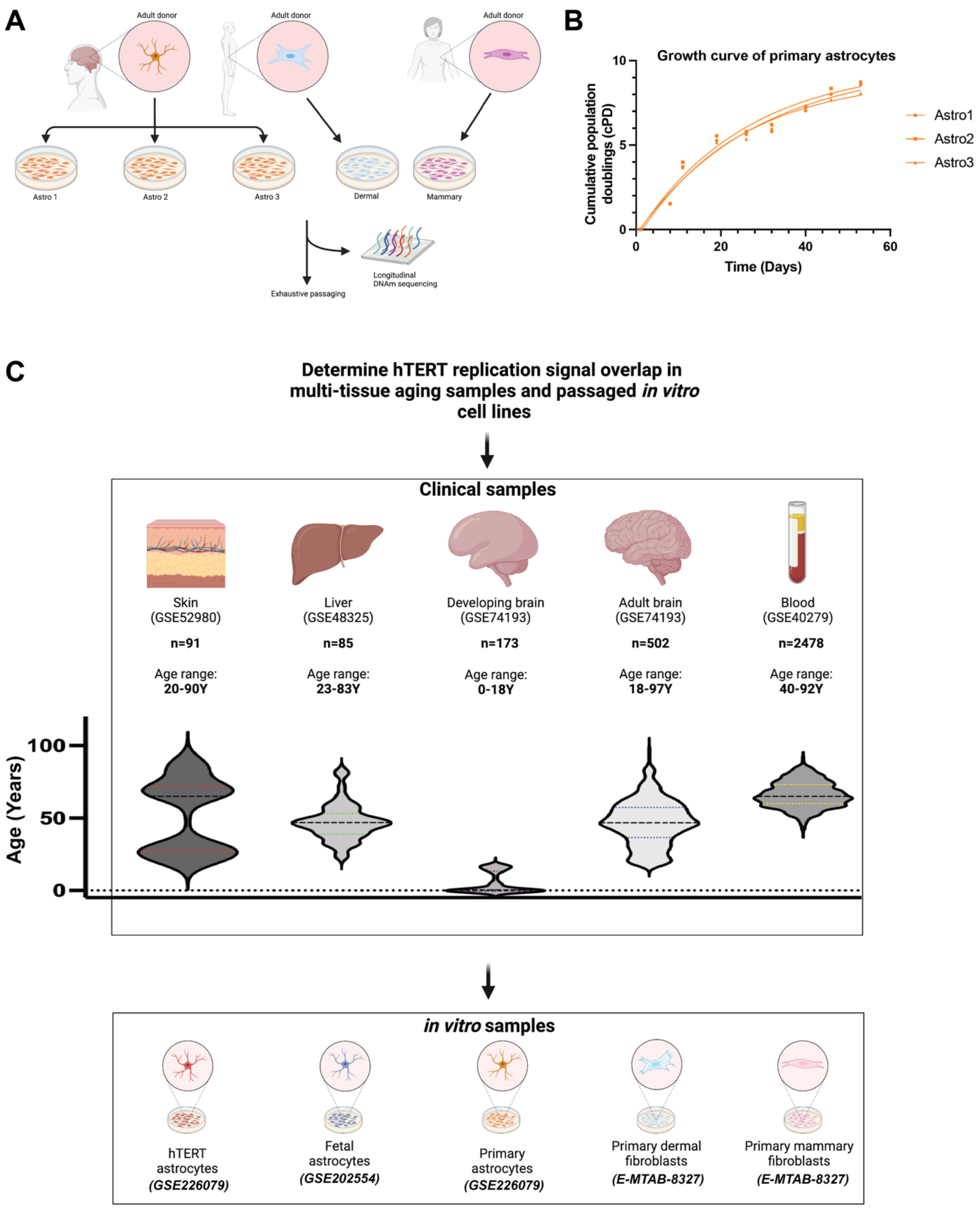
A
Adult donor
Adult donor
Adult donor
Astro 1
Astro 2
Astro 3
Dermal
Mammary
Exhaustive passaging
Longitudinal DNAm sequencing
B
Growth curve of primary astrocytes
Cumulative population doublings (cPD)
10
5
0
0
20
40
60
Time (Days)
Astro1
Astro2
Astro3
C
Determine hTERT replication signal overlap in multi-tissue aging samples and passaged in vitro cell lines
Clinical samples
Skin (GSE52980)
n=91
Age range: 20-90Y
Liver (GSE48325)
n=85
Age range: 23-83Y
Developing brain (GSE74193)
n=173
Age range: 0-18Y
Adult brain (GSE74193)
n=502
Age range: 18-97Y
Blood (GSE40279)
n=2478
Age range: 40-92Y
Age (Years)
100
50
0
in vitro samples
hTERT astrocytes (GSE226079)
Fetal astrocytes (GSE202554)
Primary astrocytes (GSE226079)
Primary dermal fibroblasts (E-MTAB-8327)
Primary mammary fibroblasts (E-MTAB-8327)

Fig. S3: Clustering input and validation data for determining module drivers. (A) Schematic displaying method of extracting the primary cell lines used in the validation analysis of this study. Note, primary dermal and mammary fibroblasts were extracted by the authors of E-MTAB-8327. **(B)** Plot displaying the cumulative population doublings of primary astrocytes exhaustively passaged 10x, which was also when growth arrest was achieved. **(C)** Summary schematic of all multi-tissue clinical data and *in vitro* data used in the training and validation of module clock measures and CellDRIFT. Note, population distribution of all clinical datasets are displayed with violin plots. The GEO accession of the data we generated in this study is accessed via GSE226079.

A

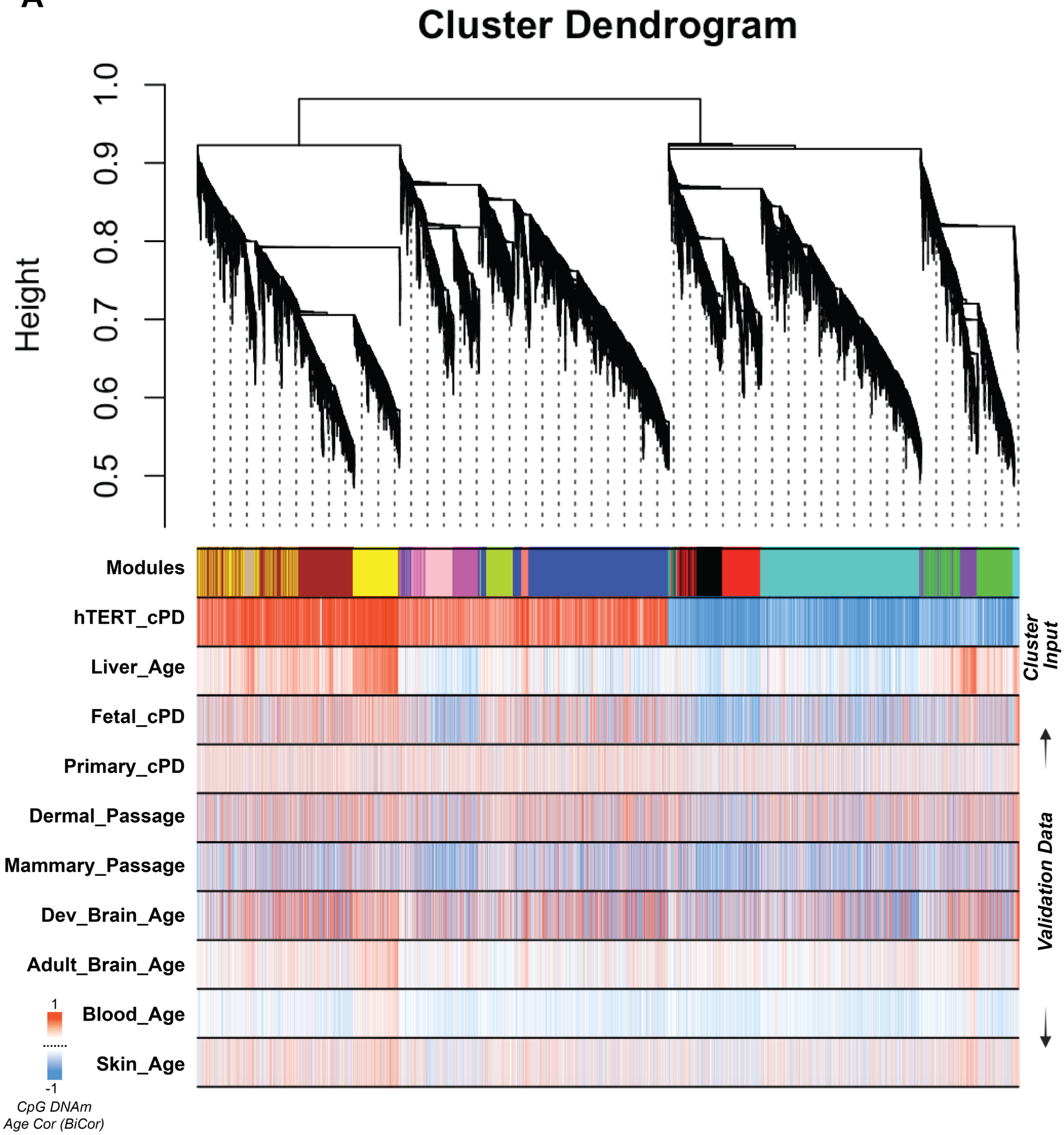
Cluster Dendrogram
Height
1.0
0.9
0.8
0.7
0.6
0.5
Modules
hTERT_cPD
Liver_Age
Fetal_cPD
Primary_cPD
Dermal_Passage
Mammary_Passage
Dev_Brain_Age
Adult_Brain_Age
Blood_Age
Skin_Age
Cluster Input
Validation Data
1
-1
CpG DNAm
Age Cor (BiCor)

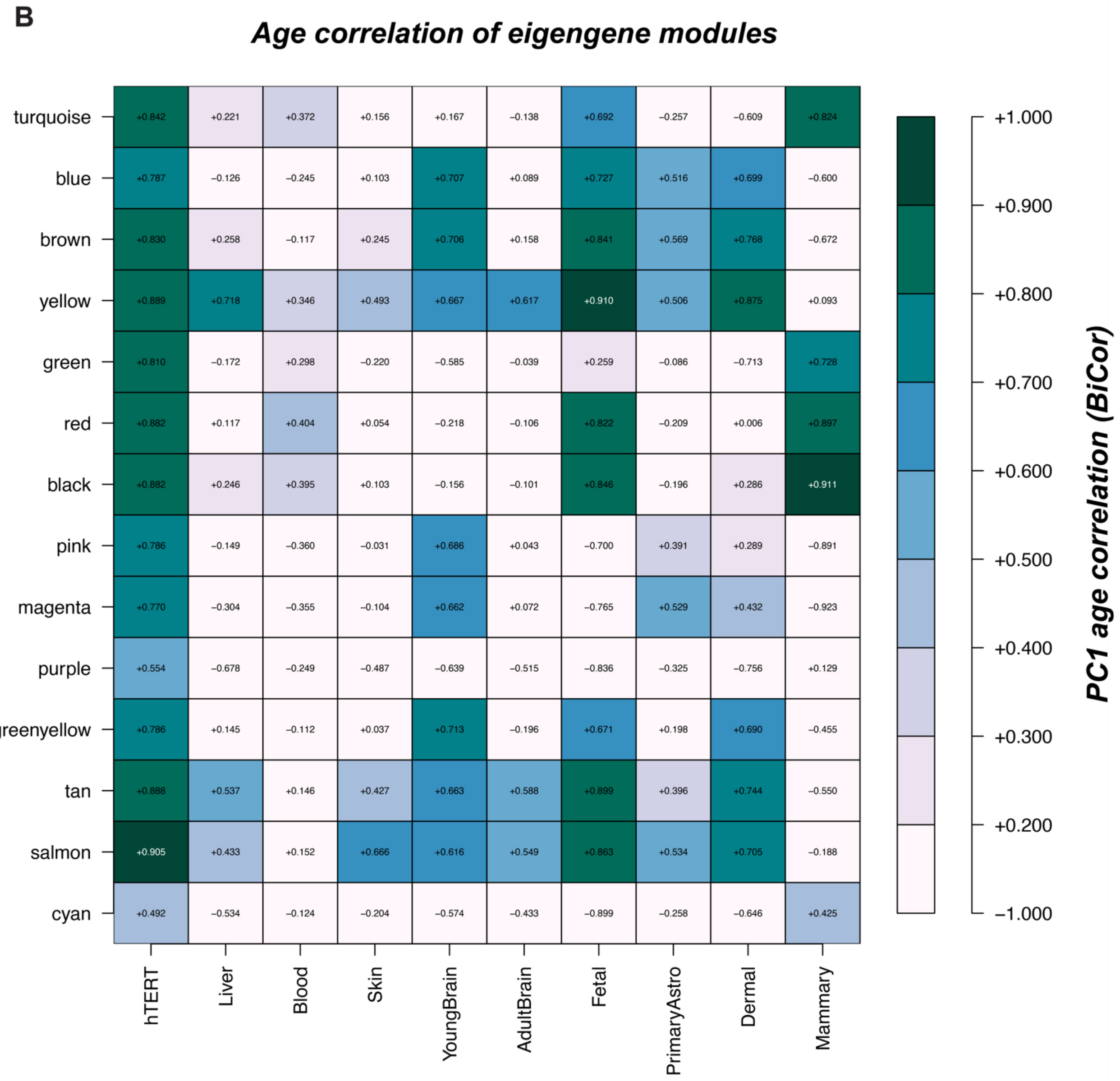

Fig. S4: Multi-tissue and *in vitro* assessments of module relevance. (A) Cluster dendrogram with all input (hTERT + liver aging) and validation *in vitro* and *in vivo* samples. Plot labels display the BirCor correlations (cPD, Age in years or Passage) for all 20,101 CpGs used in the clustering analysis. **(B)** Eigengene age correlation of module CpGs in multi-tissue and *in vitro* validation datasets. PC1 validation of module CpGs analyzed in relation to hTERT validation directionality.

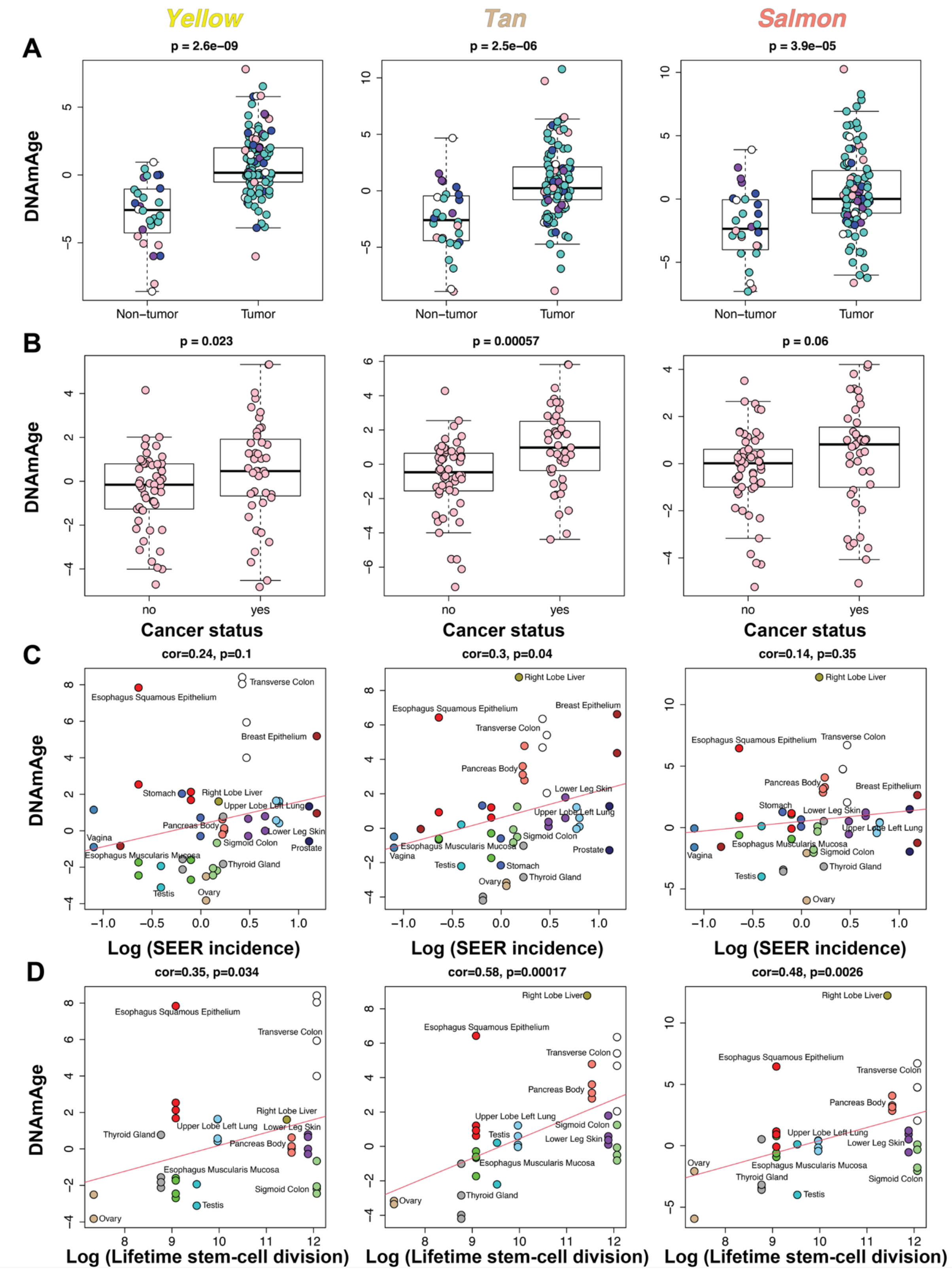
Yellow
Tan
Salmon
A
p = 2.6e−09
p = 2.5e−06
p = 3.9e−05
DNAmAge
Non−tumor
Tumor
B
p = 0.023
p = 0.00057
p = 0.06
no
yes
Cancer status
C
cor=0.24, p=0.1
cor=0.3, p=0.04
cor=0.14, p=0.35
Log (SEER incidence)
D
cor=0.35, p=0.034
cor=0.58, p=0.00017
cor=0.48, p=0.0026
Log (Lifetime stem-cell division)
Esophagus Squamous Epithelium
Transverse Colon
Breast Epithelium
Right Lobe Liver
Stomach
Upper Lobe Left Lung
Pancreas Body
Lower Leg Skin
Vagina
Sigmoid Colon
Prostate
Esophagus Muscularis Mucosa
Thyroid Gland
Testis
Ovary

Fig. S5: Physiological PC clocks (DNAmYellow/DNAmTan/DNAmSalmon) analysis in cancer and whole-body tissue datasets. (A) Pooled cancer and normal tissue from breast, colon, lung, pancreas and thyroid cancer patients and controls, evaluated via DNAmYellow, DNAmTan and DNAmSalmon module clocks for DNAm acceleration in cancer tissue (GSE53051). Teal=Thyroid, Pink=Breast, White=Lung, Purple=Pancreas and Blue=Colon. DNAmAge scores were residualized by age, sex and tissue type. **(B)** Differences in module clocks epigenetic risk in healthy breast tissue of known breast cancer patients and participants with no history of prior breast cancer. DNAmAge scores were residualized by age, menopause status and BMI prior to analysis. **(C)** Whole-body DNAmAge analysis from module clocks in relation to propensity for cancer (SEER incidence per 100,000 people) of 14 different tissues from 4 individuals (ENTEx study). **(D)** Plot showing correlation between lifetime stem cell divisions and DNAmAge from module clocks in non-zero cancer risk tissues from C. Only tissues with reported lifetimes stem cell division from Vogelstein et. al 2015 [4] were analyzed.

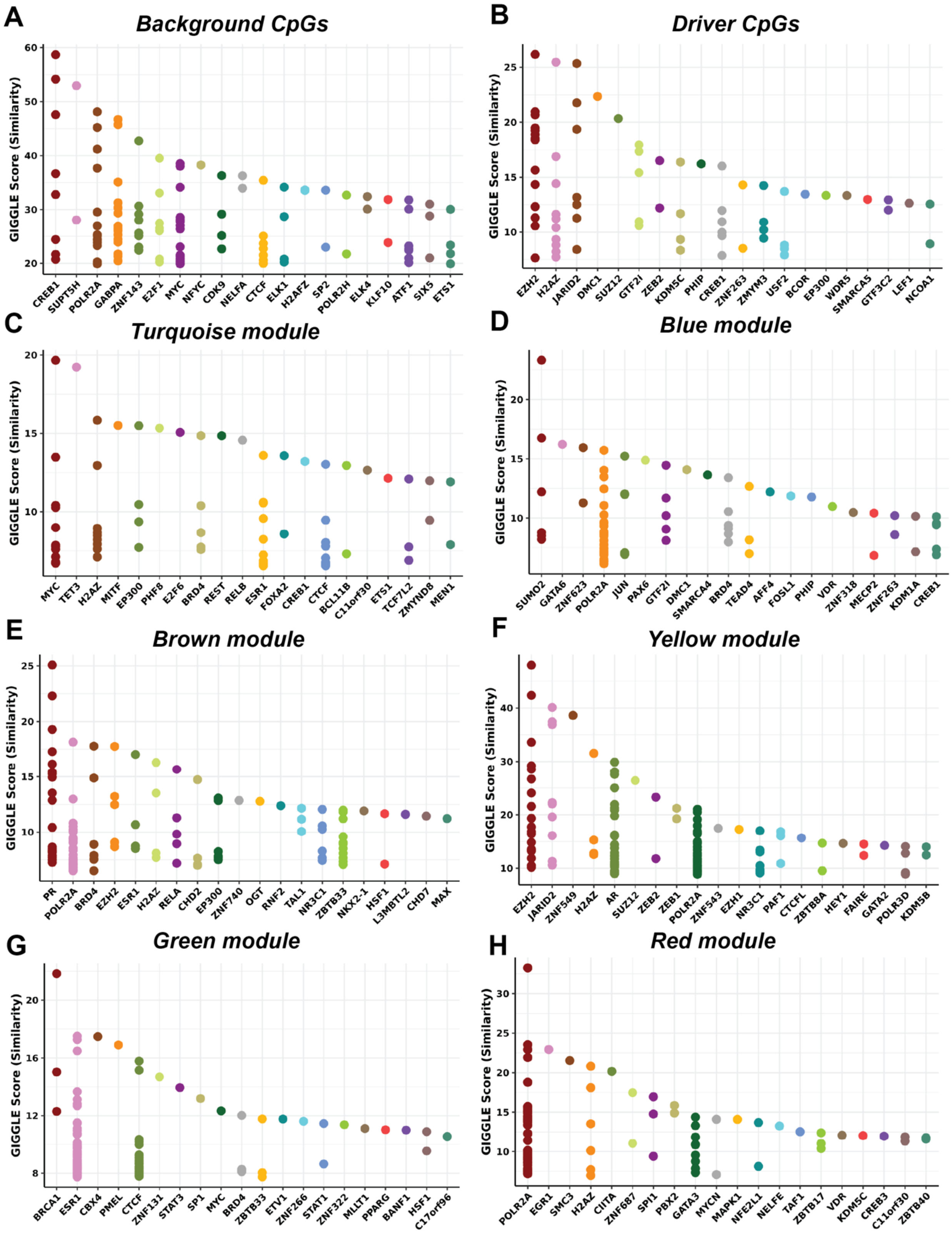
A
Background CpGs
GIGGLE Score (Similarity)
60
50
40
30
20
CREB1 SUPT5H POLR2A GABPA ZNF143 E2F1 MYC NFYC CDK9 NELFA CTCF ELK1 H2AFZ SP2 POLR2H ELK4 KLF10 ATF1 SIX5 ETS1
B
Driver CpGs
GIGGLE Score (Similarity)
25
20
15
10
EZH2 H2AZ JARID2 DMC1 SUZ12 GTF2I ZEB2 KDM5C PHIP CREB1 ZNF263 ZMYM3 USF2 BCOR EP300 WDR5 SMARCA5 GTF3C2 LEF1 NCOA1
C
Turquoise module
GIGGLE Score (Similarity)
20
15
10
MYC TET3 H2AZ MITF EP300 PHF8 E2F6 BRD4 REST RELB ESR1 FOXA2 CREB1 CTCF BCL11B C11orf30 ETS1 TCF7L2 ZMYND8 MEN1
D
Blue module
GIGGLE Score (Similarity)
20
15
10
SUMO2 GATA6 ZNF623 POLR2A JUN PAX6 GTF2I DMC1 SMARCA4 BRD4 TEAD4 AFF4 FOSL1 PHIP VDR ZNF318 MECP2 ZNF263 KDM1A CREB1
E
Brown module
GIGGLE Score (Similarity)
25
20
15
10
PR POLR2A BRD4 EZH2 ESR1 H2AZ RELA CHD2 EP300 ZNF740 OGT RNF2 TAL1 NR3C1 ZBTB33 NKX2-1 HSF1 L3MBTL2 CHD7 MAX
F
Yellow module
GIGGLE Score (Similarity)
40
30
20
10
EZH2 JARID2 ZNF549 H2AZ AR SUZ12 ZEB2 ZEB1 POLR2A ZNF543 EZH1 NR3C1 PAF1 CTCFL ZBTB8A HEY1 FAIRE GATA2 POLR3D KDM5B
G
Green module
GIGGLE Score (Similarity)
20
16
12
8
BRCA1 ESR1 CBX4 PMEL CTCF ZNF131 STAT3 SP1 MYC BRD4 ZBTB33 ETV1 ZNF266 STAT1 ZNF322 MLLT1 PPARG BANF1 HSF1 C17orf96
H
Red module
GIGGLE Score (Similarity)
30
25
20
15
10
POLR2A EGR1 SMC3 H2AZ CIITA ZNF687 SPI1 PBX2 GATA3 MYCN MAPK1 NFE2L1 NELFE TAF1 ZBTB17 VDR KDM5C CREB3 C11orf30 ZBTB40

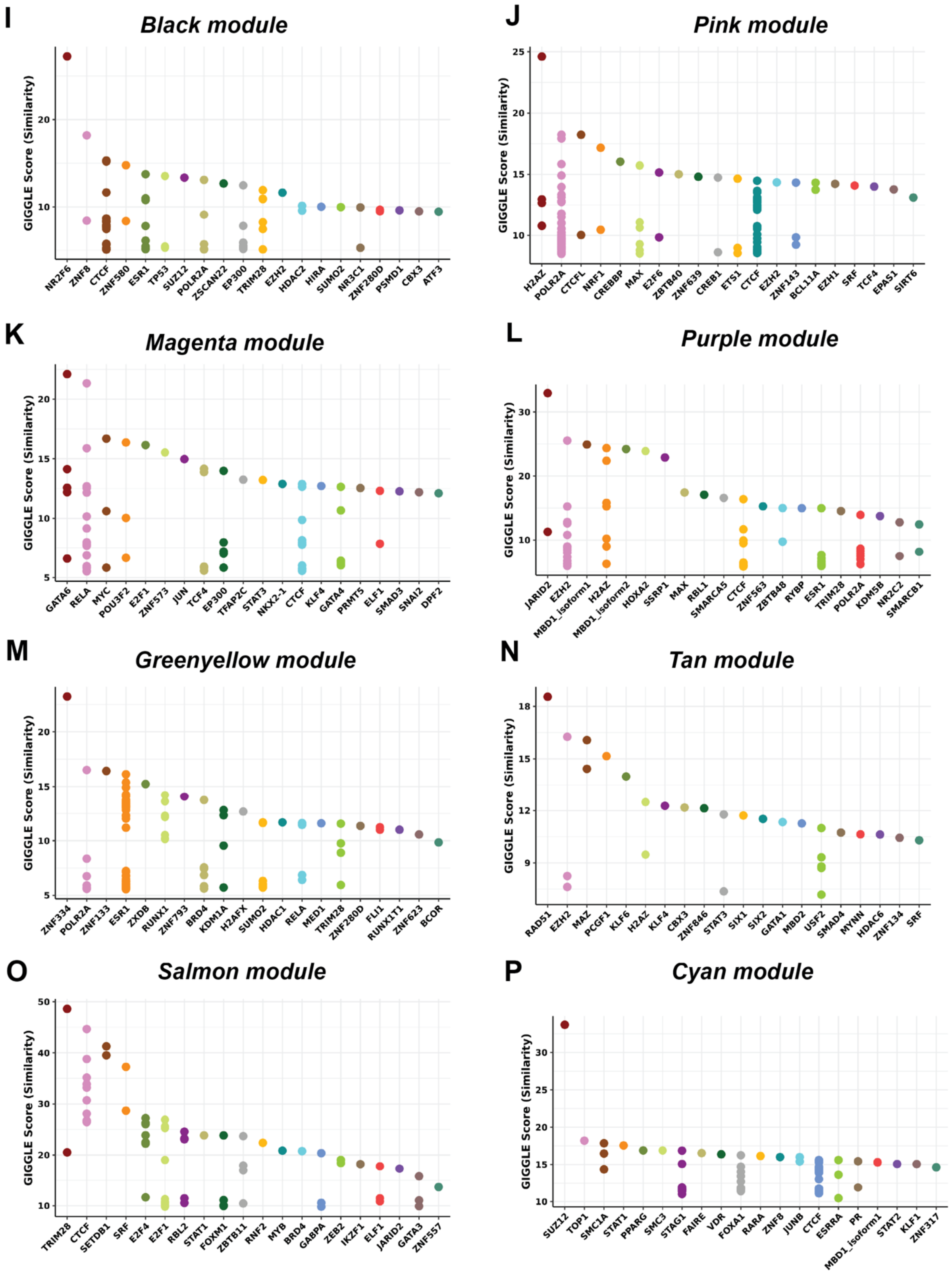

I
Black module
GIGGLE Score (Similarity)
20
10
NR2F6 ZNF8 CTCF ZNF580 ESR1 TP53 SUZ12 POLR2A ZSCAN22 EP300 TRIM28 EZH2 HDAC2 HIRA SUMO2 NR3C1 ZNF280D PSMD1 CBX3 ATF3
J
Pink module
GIGGLE Score (Similarity)
25
20
15
10
H2AZ POLR2A CTCFL NRF1 CREBBP MAX E2F6 ZBTB40 ZNF639 CREB1 ETS1 CTCF EZH2 ZNF143 BCL11A EZH1 SRF TCF4 EPAS1 SIRT6
K
Magenta module
GIGGLE Score (Similarity)
20
15
10
5
GATA6 RELA MYC POU3F2 E2F1 ZNF573 JUN TCF4 EP300 TFAP2C STAT3 NKX2-1 CTCF KLF4 GATA4 PRMT5 ELF1 SMAD3 SNAI2 DPF2
L
Purple module
GIGGLE Score (Similarity)
30
20
10
JARID2 EZH2 MBD1_isoform1 H2AZ MBD1_isoform2 HOXA2 SSRP1 MAX RBL1 SMARCA5 CTCF ZNF563 ZBTB48 RYBP ESR1 TRIM28 POLR2A KDM5B NR2C2 SMARCB1
M
Greenyellow module
GIGGLE Score (Similarity)
20
15
10
5
ZNF334 POLR2A ZNF133 ESR1 ZXDB RUNX1 ZNF793 BRD4 KDM1A H2AFX SUMO2 HDAC1 RELA MED1 TRIM28 ZNF280D FLI1 RUNX1T1 ZNF623 BCOR
N
Tan module
GIGGLE Score (Similarity)
18
15
12
9
RAD51 EZH2 MAZ PCGF1 KLF6 H2AZ KLF4 CBX3 ZNF846 STAT3 SIX1 SIX2 GATA1 MBD2 USF2 SMAD4 MYNN HDAC6 ZNF134 SRF
O
Salmon module
GIGGLE Score (Similarity)
50
40
30
20
10
TRIM28 CTCF SETDB1 SRF E2F4 E2F1 RBL2 STAT1 FOXM1 ZBTB11 RNF2 MYB BRD4 GABPA ZEB2 IKZF1 ELF1 JARID2 GATA3 ZNF557
P
Cyan module
GIGGLE Score (Similarity)
30
25
20
15
10
SUZ12 TOP1 SMC1A STAT1 PPARG SMC3 STAG1 FAIRE VDR FOXA1 RARA ZNF8 JUNB CTCF ESRRA PR MBD1_isoform1 STAT2 KLF1 ZNF317

Q

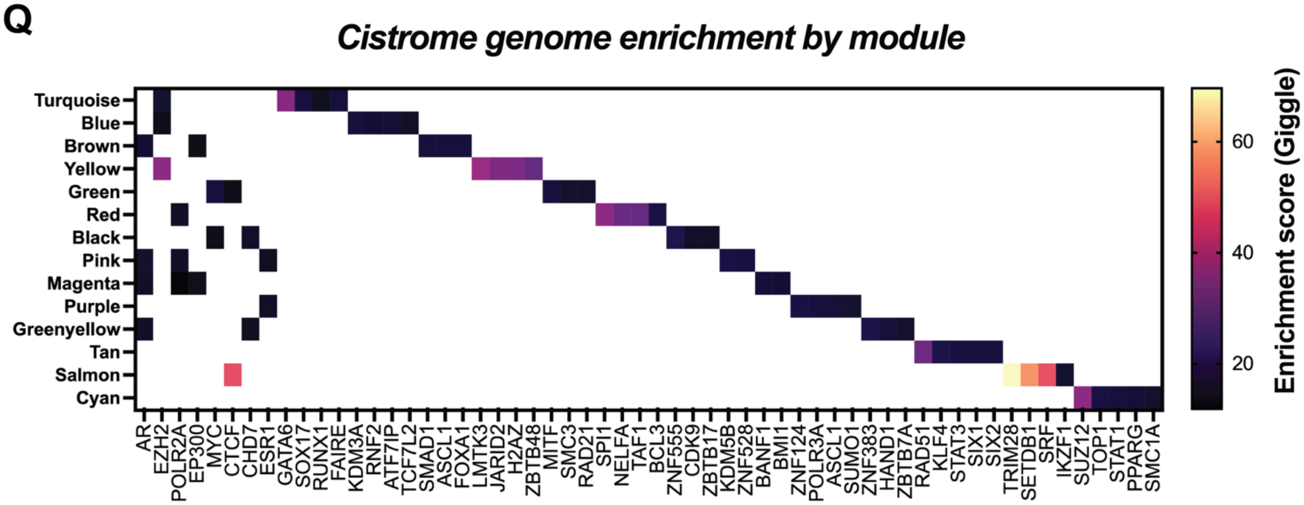

Fig. S6: Cistrome analysis of CpGs from each module. (A-P) Cistrome genome enrichment plots displaying known chromatin regulators and transcription factor sites that interact with each module. All CpGs from each module were included in the analysis. **(Q)** Summary plot of top 5 enriched genes for each module. Note, the summary module enrichment analysis used the top 100 CpGs from each module, determine from the kME score. Enriched genes were normalized by selecting 100 background CpGs from the original 440k training dataset and correcting for each GSM_IDs Giggle score. Enrichment analysis is displaying the average Giggle score across all GSM_IDs, with the top 5 for each module plotted. Giggle score is a rank of genome significance between genomic locations of query file and thousands of genome files from databases like ENCODE.

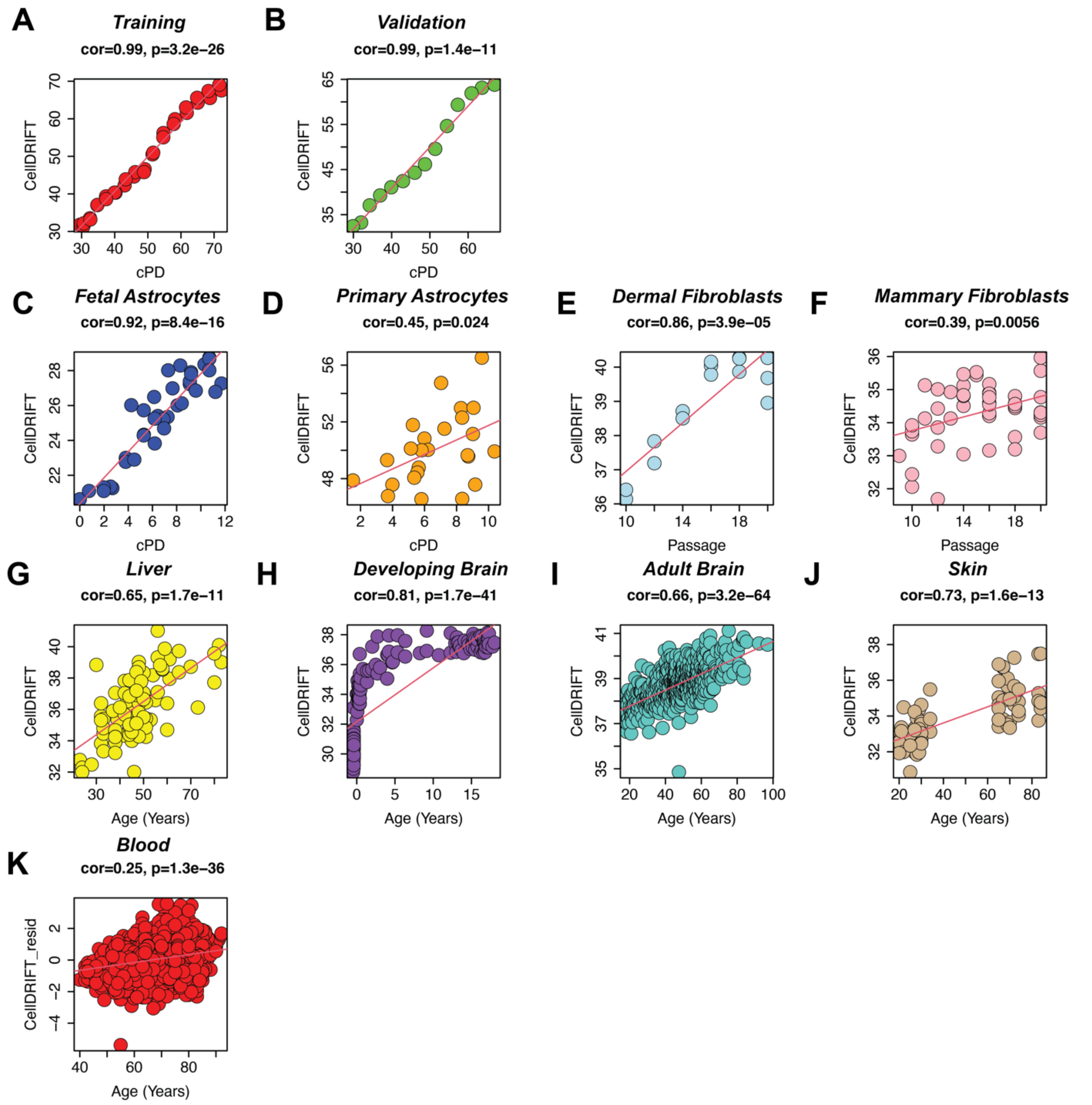

Fig. S7: Training and validation of CellDRIFT. (A) Training and **(B)** Validation of PC measure CellDRIFT, trained from yellow and tan module CpGs (2,322 total) and hTERT immortalized replication data. More training information is available in the Methods. *In vitro* validation **(C-F)** and *in vivo* multi-tissue validation **(G-K)**. Note, blood was residualized by cellular composition (b-cells, granulocytes, CD8T cells and monocytes). Age correlations and statistical significance was determined via Pearson correlations.

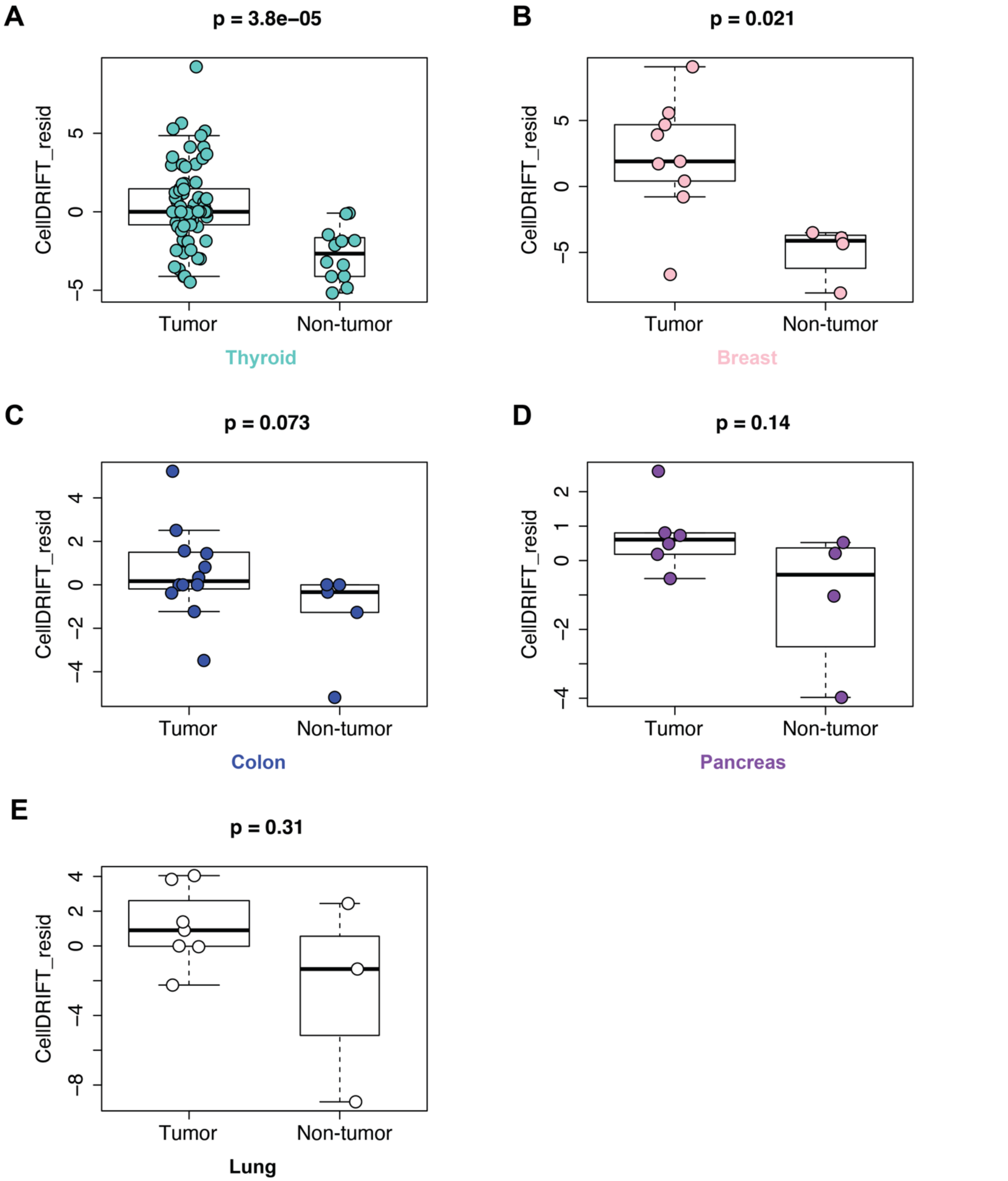

Fig. S8: Tissue-specific CellDRIFT cancer analysis from breast, colon, lung, pancreas and thyroid cancer patients and controls. Tissue specific CellDRIFT cancer analysis vs. control in thyroid **(A)**, breast **(B)**, colon **(C)**, pancreas **(D)**, and lung **(E)** tissue from GSE53051.

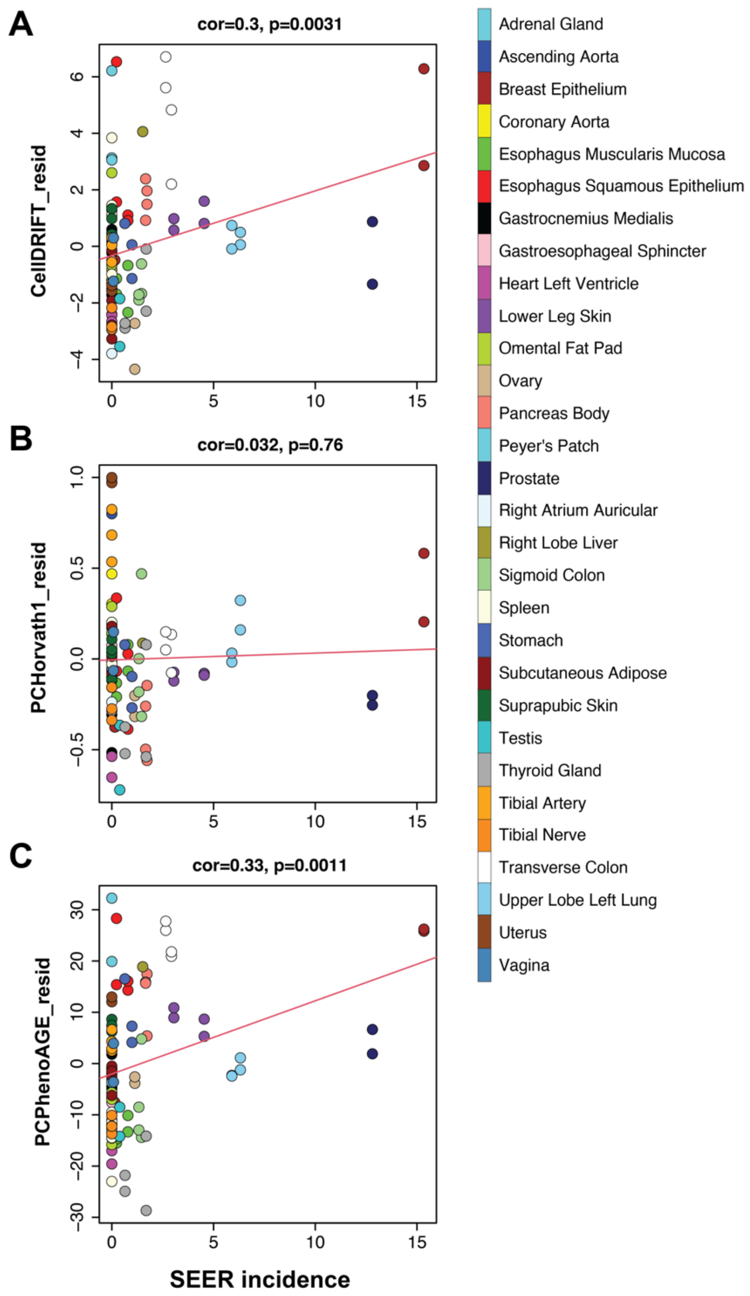

Fig. S9: Complete whole-body tissue dataset inclusive of near zero risk cancer tissues. DNAmAge analysis of **(A)** CellDRIFT**, (B)** PCHorvath1 and **(C)** PCPhenoAGE in relation to SEER incidence of 29 tissues from 4 healthy donors. All DNAmAge scores were residualized by age.

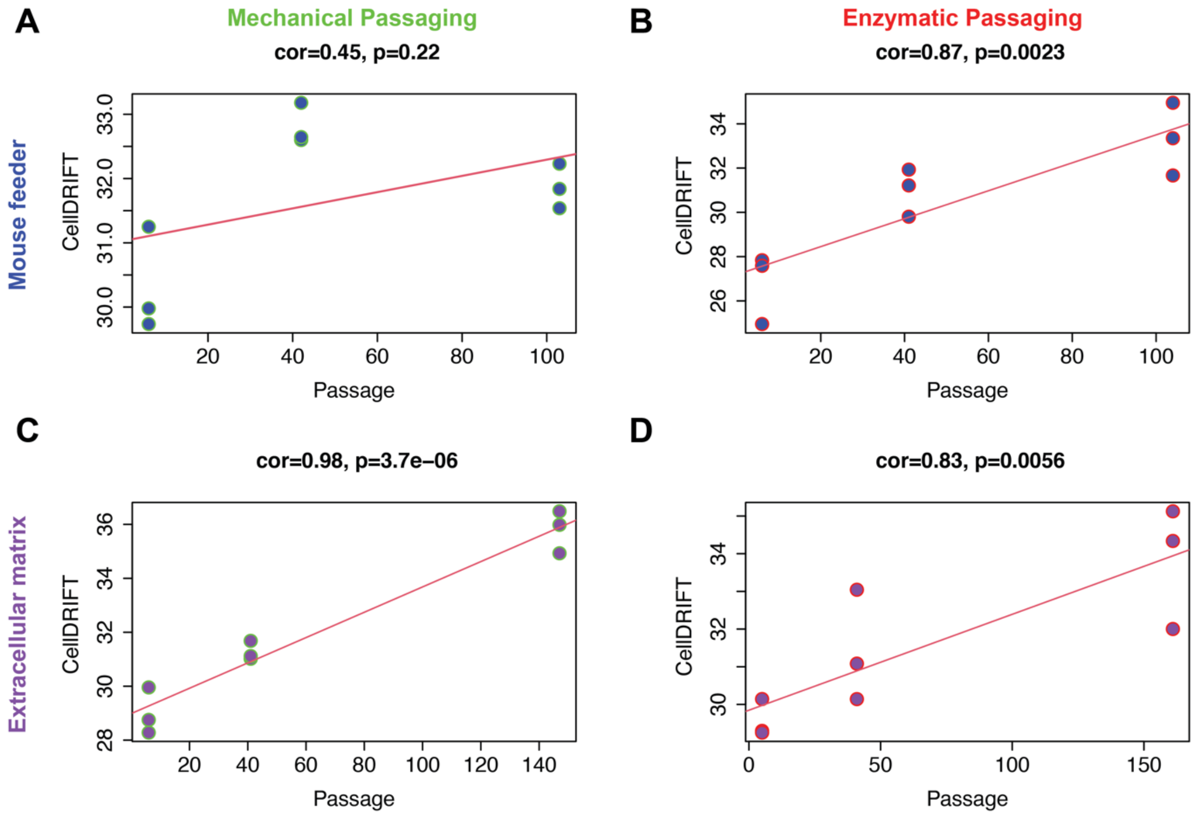

Fig. S10: CellDRIFT evaluation in extended human embryonic stem cell (hESC) passaging dataset with varying culture conditions. WA09 human embryonic stem cells (hESCs) were cultured on irradiated mouse embryonic fibroblasts (mouse feeder) or extracellular matrix and were passaged mechanically (colonies were cut into small pieces using a 18G needle) or enzymatically dissociated with Accutase (GSE56851). Cells were passaged for over two years. All cultures started from a source culture that was passaged for 37 passages. Additional information can be found from the authors (Garitaonandia et. al 2015) [42]. CellDRIFT was analyzed by growth condition (mouse feeder (**A**/**B**) or extracellular matrix (**C**/**D**)) and passaging type (mechanical (**A**/**C**) or enzymatic (**B**/**D**)).

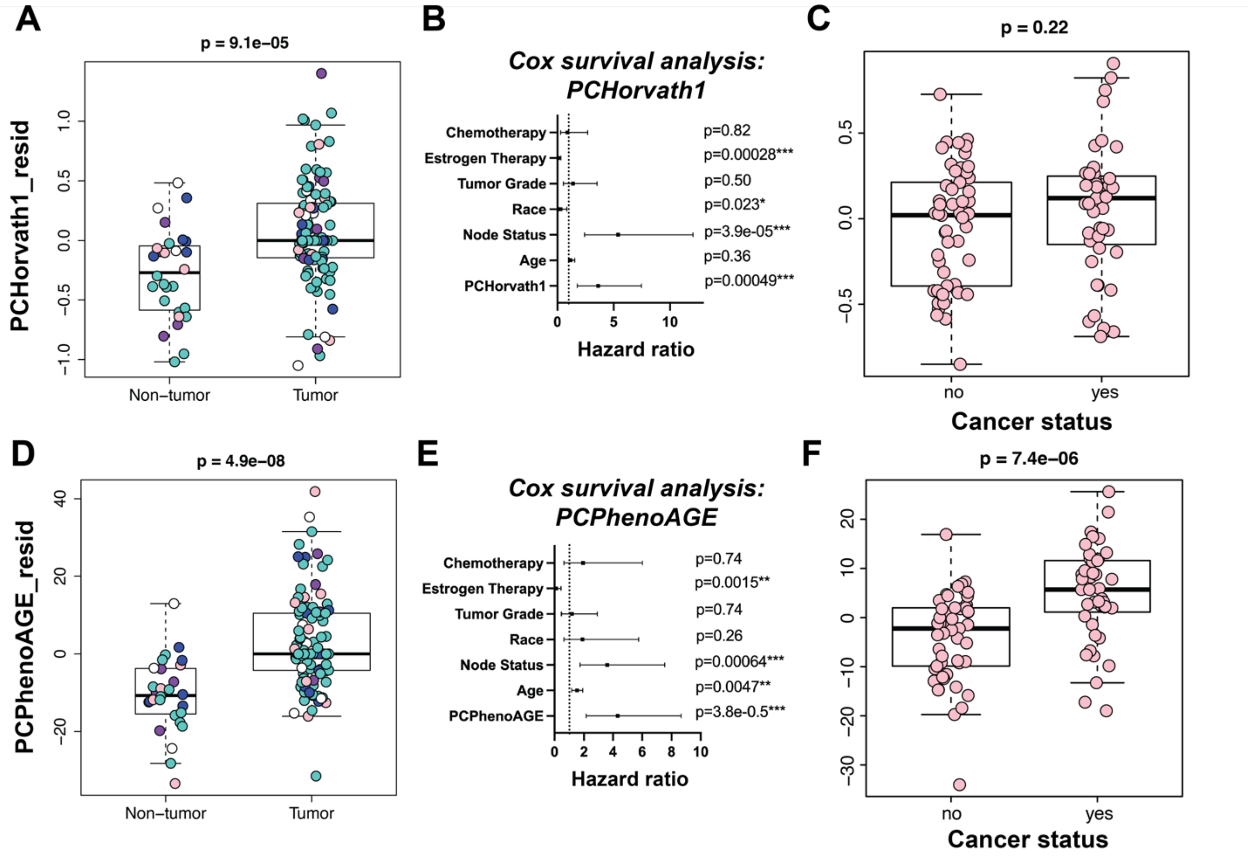

Fig. S11: Cancer detection using classically trained *ex vivo* clocks. Plots displaying cancer findings of PCHorvath1 and PCPhenoAGE measures. **(A,D)** Pooled cancer detection of breast, colon, lung, thyroid and pancreas cancer samples from GSE53051. Pink=Breast, Teal=Thyroid, White=Lung, Blue=Colon, Purple=Pancreas. DNAmAge scores were residualized by age, sex and tissue type. **(B,E)** COX survival analysis of breast cancer patients from GSE37754. Note, the hazard ratio for Age was calculated as a risk increase per 10 years of life and CellDRIFT was standardized by standard deviation. **(C,F)** Differences in DNAmAge in healthy breast tissue of known breast cancer patients and participants with no history of prior breast cancer (Rozenblit et. al 2022) [39]. DNAmAge scores were residualized by age, menopause status and BMI prior to analysis.

Table 1: List of 20,101 module CpGs. The following list includes the 20,101 module CpGs that were extracted as the highest contributing (highest normalized PC loading) CpGs from the initial hTERT passaging astrocyte model (Sup Fig. 2), which were subsequently clustered with liver aging data (GSE48325**)** to produce distinct module occupancy for each input CpG. All CpGs were assigned a module.
